# Enhanced Susceptibility of ADAP-Deficient Mice to *Listeria monocytogenes* Infection Is Associated With an Altered Phagocyte Phenotype and Function

**DOI:** 10.3389/fimmu.2021.724855

**Published:** 2021-09-30

**Authors:** Martha A. L. Böning, Gerald P. Parzmair, Andreas Jeron, Henning P. Düsedau, Olivia Kershaw, Baolin Xu, Borna Relja, Dirk Schlüter, Ildiko Rita Dunay, Annegret Reinhold, Burkhart Schraven, Dunja Bruder

**Affiliations:** ^1^ Infection Immunology, Institute of Medical Microbiology, Health Campus Immunology, Infectiology and Inflammation, Otto-von-Guericke University, Magdeburg, Germany; ^2^ Immune Regulation, Helmholtz Centre for Infection Research, Braunschweig, Germany; ^3^ Institute of Molecular and Clinical Immunology, Health Campus Immunology, Infectiology and Inflammation, Otto-von-Guericke University, Magdeburg, Germany; ^4^ Institute of Inflammation and Neurodegeneration, Health Campus Immunology, Infectiology and Inflammation, Otto-von-Guericke University, Magdeburg, Germany; ^5^ Department of Veterinary Medicine, Institute of Veterinary Pathology, Freie Universität, Berlin, Germany; ^6^ Experimental Radiology, Department of Radiology and Nuclear Medicine, Health Campus Immunology, Infectiology and Inflammation, Otto-von-Guericke University, Magdeburg, Germany; ^7^ Institute of Medical Microbiology and Hospital Epidemiology, Hannover Medical School, Hannover, Germany; ^8^ Institute of Medical Microbiology and Hospital Hygiene, Health Campus Immunology, Infectiology and Inflammation, Otto-von-Guericke University, Magdeburg, Germany; ^9^ Center for Behavioral Brain Sciences, Otto-von-Guericke University, Magdeburg, Germany

**Keywords:** ADAP, adaptor protein, *Listeria monocytogenes*, neutrophils, inflammatory monocytes, phagocytosis, neutrophil extracellular traps

## Abstract

The adhesion and degranulation-promoting adaptor protein (ADAP) serves as a multifunctional scaffold and is involved in the formation of immune signaling complexes. To date, only limited data exist regarding the role of ADAP in pathogen-specific immunity during *in vivo* infection, and its contribution in phagocyte-mediated antibacterial immunity remains elusive. Here, we show that mice lacking ADAP (ADAPko) are highly susceptible to the infection with the intracellular pathogen *Listeria monocytogenes* (*Lm*) by showing enhanced immunopathology in infected tissues together with increased morbidity, mortality, and excessive infiltration of neutrophils and monocytes. Despite high phagocyte numbers in the spleen and liver, ADAPko mice only inefficiently controlled pathogen growth, hinting at a functional impairment of infection-primed phagocytes in the ADAP-deficient host. Flow cytometric analysis of hallmark pro-inflammatory mediators and unbiased whole genome transcriptional profiling of neutrophils and inflammatory monocytes uncovered broad molecular alterations in the inflammatory program in both phagocyte subsets following their activation in the ADAP-deficient host. Strikingly, *ex vivo* phagocytosis assay revealed impaired phagocytic capacity of neutrophils derived from *Lm*-infected ADAPko mice. Together, our data suggest that an alternative priming of phagocytes in ADAP-deficient mice during *Lm* infection induces marked alterations in the inflammatory profile of neutrophils and inflammatory monocytes that contribute to enhanced immunopathology while limiting their capacity to eliminate the pathogen and to prevent the fatal outcome of the infection.

## Introduction

Adaptor proteins serve as scaffolds for a variety of signaling complexes and are involved in diverse signaling processes in immune cells. Among them, the adhesion and degranulation-promoting adaptor protein (ADAP) belongs to the group of cytosolic adaptor proteins (1–3). ADAP was first described and is best studied in T cells as an interaction partner for the Fyn-binding protein (Fyb), also termed SH2-domain-containing leukocyte protein of 76 kDa (SLP-76)-associated phosphoprotein of 130 kDa (SLAP-130) (4, 5). ADAP entails numerous domains, together facilitating the binding to parts of different signaling complexes, consequently being involved in cell migration, adhesion, and actin cytoskeleton re-organization in T cells (6). Next to T cells, ADAP is expressed in natural killer (NK) cells (7), platelets (8), and cells of myeloid origin (9) including neutrophils and monocytes.

For neutrophils, a crucial role of ADAP in regulating cell adhesion by integrins has been described. More specifically, ADAP knockout (ADAPko) mice are less susceptible to ischemia–reperfusion-induced acute kidney injury (AKI), which is associated with ADAP-dependent neutrophil migration in AKI (10). Furthermore, the selective loss of SLP-76 expression in myeloid cells affects neutrophil functions *in vivo* (11). While available data underline important roles of ADAP in integrin-associated adhesion and leukocyte recruitment, its role in pathogen-specific immunity during *in vivo* infection and more specifically in phagocytes remains elusive to date.

*Listeria monocytogenes* (*Lm*) is an intracellular pathogen that can cause life-threatening infections in humans and animals (12) while representing a well-established model pathogen to study different lines of innate and adaptive immunity in mice (13, 14). *Lm* infection induces a strong innate immune response, essential for host protection against the pathogen (13). Neutrophils and monocytes are rapidly recruited to sites of infection, where they are activated and exert central antimicrobial functions. Consequently, targeted depletion of these cell types enhances susceptibility to *Lm* infection in mice (12).

Next to the production of pro-inflammatory cytokines, neutrophils directly mediate antimicrobial activities by phagocytosis and intracellular killing of pathogens. In addition, they release microbicidal reactive nitrogen (NOS) and reactive oxygen species (ROS), as well as extracellular traps [neutrophil ETs (NETs)], which immobilize extracellular pathogens and promote their killing (15). During *Lm* infection, a massive influx of neutrophils to the liver results in a drop in bacterial burden (16), and moreover, the contribution of neutrophils in controlling bacterial growth during murine listeriosis was proven in antibody-mediated depletion experiments (17). Next to NETs, neutrophils are able to control bacterial pathogens by phagocytosis and subsequent intracellular killing by the production of reactive nitrogen species and ROS (18). Neutrophils play an important role in the immune response to low dose *Lm* infection in the liver, while in the spleen neutrophils play a vital role for protection against high dose infection (17). Moreover, neutrophils are more effective at phagocytosis of *Lm* than inflammatory monocytes (19). Of note, tumor necrosis factor (TNF) and especially inducible nitric oxide synthase (iNOS) are essential for the clearance of murine *Lm* infection (20, 21).

Besides neutrophils, monocytes are crucial for protection against listeriosis (21). Peripheral monocytes can be roughly subdivided into two main subsets: CX3CR1^low^CCR2^+^Ly6C^high^ inflammatory monocytes and CX3CR1^high^CCR2^−^Ly6C^low^ patrolling monocytes (22, 23). Of note, the Ly6C^high^ inflammatory monocytes are actively recruited to sites of infection (24), and monocyte recruitment is a hallmark of listeriosis. Mice deficient in CC chemokine receptor 2 (CCR2) or its ligand (CCL2) are highly susceptible to *Lm* and reach abortion criteria early after infection (25). Hence, the lack of Ly6C^high^ inflammatory monocytes is associated with a rapid death of *Lm*-infected mice, indicating their central role during listeriosis (26).

Cytokines orchestrate the host immunity during *Lm* infection (14). Mice deficient in granulocyte colony-stimulating factor (G-CSF) exhibit abnormally low numbers of neutrophils in the blood and thus show increased bacterial burdens in both the spleen and liver as compared with wild type animals (27). During infection, neutrophils produce substantial amounts of TNF-α, and consequently, neutrophil depletion in *Lm*-infected mice leads to decreased TNF-α levels and increased bacterial load (17). Accordingly, TNF-α- or TNF receptor 1 (TNFR1)-deficiency results in an increased susceptibility to *Lm* infection in mice (28). Another cytokine produced by phagocytes early on during bacterial infection is interleukin (IL)-1. Immediately after *Lm* infection, IL-1α as well as IL-1β are produced in the spleen and liver, and exogenous IL-1α is known to decrease the bacterial burden by promoting neutrophil recruitment to infection sites (29). Macrophages that serve as the primary site of *Lm* replication are responsible for the majority of pathogen elimination during the early phase of infection. They produce considerable amounts of TNF-α and IL-12, which induce the production of interferon (IFN)-γ by NK cells, thereby enabling NK cells to kill intracellular bacteria (30).

In this study, we aimed to extend the yet very limited knowledge on the roles of ADAP in phagocytes during *in vivo* infection. More specifically, we comprehensively studied the impact of ADAP-deficiency on pathogen-specific immunity, utilizing a murine model of *Lm* infection in conventional and conditional ADAP-deficient mice.

## Materials and Methods

### Animals

Conventional ADAPko and mice with floxed ADAP alleles (ADAP^fl/fl^) were described before (31, 32). To generate conditional knockout mice with deletion of ADAP in myeloid cells, ADAP^fl/fl^ were crossed with LysM-Cre knockin mice (33, 34). To investigate specific effects of ADAP deletion in myeloid cells, ADAP^fl/fl^Cre^het^ (conditional knockout) mice were used in this study, while ADAP^wt/wt^Cre^het^ animals served as littermate controls to exclude off-target effects of Cre recombinase. These mice developed normal and total leukocyte numbers, and the distribution of neutrophils and macrophages in the bone marrow and spleen did not reveal any differences between both genotypes (33). Mice were kept under specific pathogen-free conditions in environmentally controlled clean rooms and used at 9–18 weeks of age. If not otherwise stated, experiments were performed using male mice. All animal experiments were conducted according to the institutional guidelines, and the study was approved by the Niedersächsisches Landesamt für Verbraucherschutz und Lebensmittelsicherheit (33.9.42502-04-10/0256) as well as the local government agencies (Landesverwaltungsamt Sachsen-Anhalt; AZ 42502-2-1603 UniMD).

### *Listeria monocytogenes* Infection and Quantification of Bacteria in Organs

For *in vivo* infections in mice, *Lm* strain 10403S was used (35, 36). Frozen stocks of *Lm* were thawed and diluted in brain heart infusion (BHI) medium followed by an overnight growth on agar plates. One day before the infection, an overnight liquid culture (BHI) was prepared, which was diluted with fresh medium (1:5) and cultivated at 37°C for 4 h before bacteria were harvested. Bacterial numbers were determined by measuring OD600_nm_ using a spectrophotometer, and based on a previously established OD/cell-density correlation curve, the suspension was further diluted in phosphate-buffered saline (PBS) to the desired infection dose. Mice were infected by intravenous (i.v.) injection into the tail vein with bacteria suspended in 100 μl. If not stated otherwise in the figure legends, the infection dose was 2.5 × 10^4^ colony-forming units (CFU) of *Lm*. To determine the actual infectious dose, serial dilutions were plated on BHI agar followed by an overnight incubation of the plate at 37°C. The colonies were counted the next day, taking into consideration the dilution factor, showing the actual number of bacteria that had been applied to each mouse.

To determine the bacterial burden (CFU) in the spleens and livers, mice were sacrificed at indicated times, and organs were collected and homogenized in sterile tubes with 1 ml (spleen) or 2 ml (liver) of 0.2% (v/v) IGEPAL CA-630/PBS. Serial dilutions were plated on BHI agar plates, and CFU were counted after incubation (24 h, 37°C).

### Histopathological Analyses

The spleens and livers were immersion fixed with 4% buffered formalin, embedded in paraffin, sectioned at 4-μm thickness, and H&E stained. Blinded histological evaluations were performed by an expert veterinary pathologist (Dr. Olivia Kershaw, Berlin) according to reference (37).

### Cell Preparation

The spleens and livers were flushed by heart perfusion with PBS. Single-cell suspension of splenocytes was prepared as described before (38). Liver leukocytes were either isolated using the mouse liver dissociation Kit and the gentleMACS dissociator device (both from Miltenyi Biotec) according to the manufacturer’s instructions or, alternatively, minced with scissors, suspended in 3 ml of digestion medium (IMDM-complete with 0.02 mg L^−1^ of Collagenase D and 0.01 mg L^−1^ of DNAse; both from Sigma-Aldrich), transferred to Falcon tubes, and incubated (30 min, 37°C) while the suspension was sheared frequently. After 30 min, 2 ml of fresh digestion medium was added, and the livers were incubated for an additional 15 min. Afterwards, 60 μl of 0.5 mol L^−1^ ethylenediaminetetraacetic acid (EDTA) was added, and samples were rested (5 min, 37°C) to stop enzymatic digestion. Cell suspension was passed through 100-μm cell strainers, washed with PBS, and afterwards passed through a 70-μm cell strainer, followed by centrifugation (300 rcf, 10 min) at room temperature (RT). Erythrocyte lysis was performed as described previously (38). The resulting pellet was resuspended in 10 ml of 35% EasyColl/PBS and centrifuged (1,800 rpm, 10 min, RT, no brake). Leukocyte pellet was resuspended in IMDM-complete and stored on ice.

### Flow Cytometry

For flow cytometric analyses, single-cell suspensions were first stained with anti-CD16/32 (Fc-block, BioLegend) to prevent unspecific antibody binding. The panel used to determine infiltrating cells (absolute cell numbers) in the spleens and livers was the following: live/dead discrimination (live/dead Fixable Blue, BioLegend), anti-CD11b (Pacific Blue, M1/70, BioLegend), anti-Ly6G (PE-Cy7, 1A8, BioLegend), and anti-Gr-1 (PerCP-Cy5.5, RB6-8C5, BioLegend). Fixation was performed with 2% (v/v) paraformaldehyde (PFA)/PBS (20 min, RT in dark). After being washed, cells were resuspended in PBS and stored at 4°C in the dark until further use. Neutrophils were defined as CD11b^+^Gr-1^+^Ly6G^+^ and monocytes as CD11b^+^Gr-1^+^Ly6G^−^. Flow cytometric analysis was performed using the BD LSRFortessa.

For intracellular cytokine staining of neutrophils and inflammatory monocytes, cell suspensions were stimulated for 4 h at 37°C with phorbol myristate acetate (PMA; 20 ng L^−1^, Sigma-Aldrich) and ionomycin (1 μg L^−1^, Sigma-Aldrich) in medium (IMDM-complete). After 1 h, Brefeldin A (BioLegend) and Monensin (BioLegend) were added. Cells were stained with anti-CD16/32 and for live/dead discrimination with a Fixable Viability Dye (eFlour780, eBioscience), followed by staining with the following antibodies: anti-CD4 (FITC), RM4-4, BioLegend), anti-CD8 (FITC, 53-6.7, BioLegend), and anti-B220 (FITC, RA3-6B2, BioLegend) used for cell exclusion, and anti-CD45 (PerCP-Cy5.5, 30-F11, BioLegend), anti-CD11b (BV605, M1/70, BioLegend), anti-Ly6C (APC), HK1.4, BioLegend), anti-Ly6G (PE-Cy5, 1A8, Invitrogen), and anti-CX3CR1 (BV510, SA011F11, BioLegend). Afterwards, cells were fixed with the Fixation/Permeabilization Kit (Thermo Fisher Scientific), followed by intracellular staining: anti-IL-1α (PE, ALF-161, BioLegend), anti-TNF-α (PE-eFlour610, MP6-XT22, Invitrogen), and anti-NOS2 (PE-Cy7, CXNFT, Invitrogen) for 30 min at 4°C. Cells were analyzed using an Attune NxT Flow Cytometer (Thermo Fisher Scientific). Partially representative mean cytokine positive cells (in %) and mean fluorescence intensity (MFI; geometric mean) of pooled mice per genotype are shown for intracellular staining. All data were analyzed using FlowJo software (Version 9.9.6.).

### Phagocytosis Assay

Splenic and hepatic leukocytes were placed in triplicates into 96‐well plates (4 × 10^5^ cells/well) and incubated for 2 h at 37°C (5% CO_2_). Cells incubated at 4°C served as negative controls (ctr.). Subsequently, FITC‐fluorescent, carboxylated latex beads (FluoSpheres™, F8823, Fisher Scientific) were added (cell:bead ratio of 1:5). Afterwards, cells were washed and stained using the antibody panel to identify neutrophils and inflammatory monocytes as described above. Phagocytosis of latex beads was evaluated by determining the MFI of all FITC-positive neutrophils and inflammatory monocytes according to references (39, 40).

### Quantification of Cytokines in Serum Samples

Mice were sacrificed, and blood was obtained by heart puncture. Sample preparation was performed as described (38). Serum cytokines were quantified in 1:2 diluted samples using a cytometric bead array (LEGENDplex™ Mouse Inflammation cytokine panel (13-plex), BioLegend) according to the manufacturer’s recommendations.

Analysis of TGF-β levels, measured by ELISA (BioLegend), required platelet-free plasma (PFP). This was prepared by taking 400 μl of cardiac blood and immediately mixing it with 100 μl of 0.105 mol L^−1^ sodium citrate to prevent coagulation. The stabilized blood was centrifuged (400 rcf, 5 min, 4°C), and the platelet-rich plasma (PRP) was carefully transferred to a fresh Eppendorf tube. The PRP was centrifuged again (6,800 rcf, 10 min, 4°C) to pellet the platelets. The resulting PFP was carefully transferred to a fresh Eppendorf tube and stored at −70°C until further use.

### Microarray Analyses

Neutrophils and inflammatory monocytes were sorted by fluorescence-activated cell sorting (FACS) from the spleen and liver leukocytes from *Lm*-infected mice 3 days post infection (p.i.). RNA was isolated using the RNeasy Mini Kit (Qiagen) according to the manufacturer’s instructions. To acquire a representative mix of all individual samples (n = 6 mice/genotype), sorted cells from two mice per genotype were pooled. Each genotype was analyzed in triplicates. Only neutrophils from wild-type mice were analyzed in duplicate. The preparation of samples (amplification, fragmentation, microarray hybridization, staining, and scanning) was performed at the Genome Analytics Group at Helmholtz Centre for Infection Research, Braunschweig, Germany. Samples were analyzed with the Affymetrix Clariom S mouse microarray resulting in 23 microarrays. Microarray raw data were initially analyzed using the Transcriptome Analysis Console (TAC) software (Thermo Fisher Scientific). Briefly, data were summarized, log_2_-transformed, and quantile-normalized with SST-RMA algorithm. A percentile filter, to decimate lowly abundant transcripts, was applied to the microarray data, removing transcripts with signal intensities consistently below the 20th percentile of the average signal intensity (SI) distribution in all microarrays of a given cell subset. Differential expression between ADAPko and wild type cells was calculated based on eBayes ANOVA method with false discovery rate (FDR) <0.05 and fold change (FC) cutoff of −3 < FC > + 3. Volcano plots were generated in Python using Matplotlib library and the Spyder programming environment. K-means clustering of z-score transformed normalized log_2_ signal intensities of differentially regulated genes was calculated and plotted using Genesis software (41). Gene Ontology (GO) enrichment analysis was performed with Cytoscape software and ClueGO plugin (42), using two-sided hypergeometric test with Bonferroni-step-down multiple-hypothesis-testing correction and GO-term fusion/grouping options applied. Only GO terms with FDR <0.05 and GO level ≥8 were considered. Enriched GO terms were mapped to previously determined k-means clusters, and the percentages of transcripts in each cluster affiliating to significantly enriched GO terms were color-coded and hierarchically clustered in the Genesis software. Microarray data were deposited in National Center for Biotechnology Information’s (NCBI’s) Gene Expression Omnibus (GEO) and are accessible through the GEO series accession number GSE175993.

### Staining of Neutrophil Extracellular Traps in Mouse Tissue

After euthanasia of mice and blood removal, the heart was perfused with 20 ml of PBS and afterwards with 10% formalin. The spleens and livers had been fixed *in situ* by perfusion with 4% formalin. Following this, tissues were removed and fixed in 4% formalin for 15–20 h. Tissues were then stored in 70% ethanol (dark, 4°C), paraffin-embedded, and subsequently cut into 4-μm sections as described before (43). Antigen retrieval protocol, described by Becker et al. (43), was run at 50°C for 90 min. Paraffin sections were dried, followed by dewaxing and rehydration. Subsequently, sections were incubated with heat-induced epitope retrieval (HIER) buffer (Aptum) at different temperatures before three rinsing steps with de-ionized water and one time with PBS. After antigen retrieval, sections were permeabilized for 5 min with 0.5% Triton X-100 (Sigma) in PBS at RT, followed by three rinsing steps with PBS. Sections were treated with blocking buffer, containing 2% donkey serum, 5% normal goat, 0.05% TWEEN20, and 0.05% Triton X-100 for 30 min to prevent non-specific binding before rinsing steps occurred. Primary antibodies for anti-neutrophil elastase (anti-NE; Abcam) and anti-histone H3 (Thermo Fisher) were diluted in blocking buffer incubated on the sections overnight followed by different rinsing steps. The same procedure was followed for the secondary antibodies conjugated to Alexa Fluor 488 donkey anti-rabbit IgG (Bioss) and Alexa Fluor 647 goat anti-rat IgG (Thermo Fisher) with an incubation of 30 min at RT in the dark. Staining of the nuclei was done using DAPI (Carl Roth) prepared in PBS followed by rinsing steps. Since *Lm* infection might not be homogeneously distributed throughout the organ, four pictures per slide for all three layers (12 in total) at a magnification of 200-fold were taken. Tissue sections were analyzed with the KEYENCE BZ-X800 microscope, and analysis was done by counting the number of histones and NE expression was quantified by using Image-Pro Plus 6.

### Statistics

Statistical analyses were performed using the GraphPad Prism Software version 9 (GraphPad Software, Inc., La Jolla, CA, USA).

## Results

### ADAP-Deficiency Renders Mice Highly Susceptible to Infection With the Intracellular Pathogen *Listeria monocytogenes*

**Figure 1:**
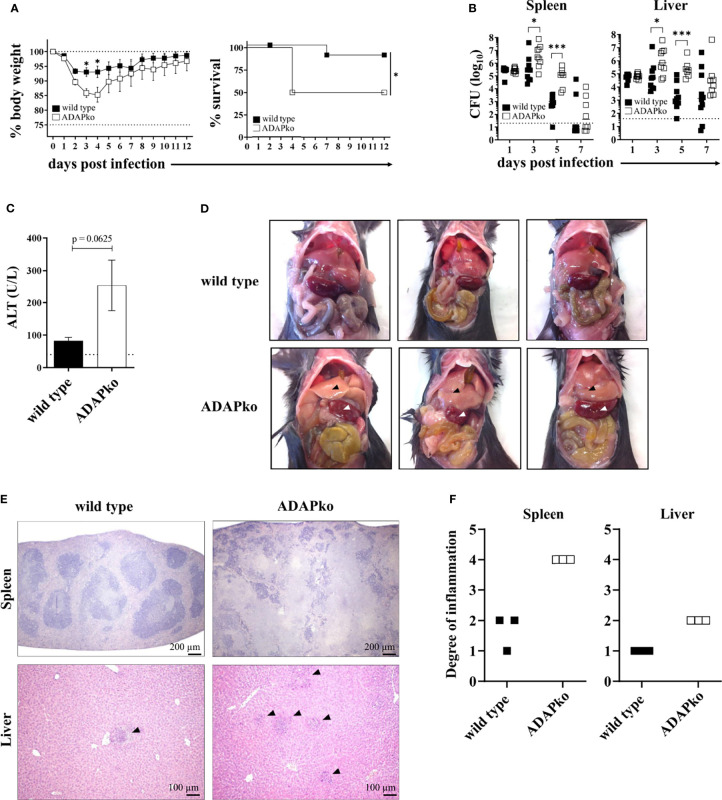
ADAP-deficiency renders mice highly susceptible to infection with the intracellular pathogen *Listeria monocytogenes* (*Lm*). Wild type (▪) and ADAPko (▫) mice were either infected i.v. with **(A)** 5 × 10^4^ CFU, **(B, C, E, F)** 2.5 × 10^4^ CFU, and **(D)** 1 × 10^4^ CFU *Lm* (strain 10403S) or were left untreated (uninfected control mice, day 0) and were sacrificed at the indicated times post infection. **(A)** Infected mice were weighed and monitored daily, and the survival was reported. Data are depicted as mean ± SEM for n = 8–14 individually analyzed mice. Statistical analyses were performed using two-way ANOVA with Bonferroni’s post-hoc test for the weight data and Mantel–Cox log-rank test for the survival data. **(B)** Bacterial loads in the spleen and liver after *Lm* infection. The dashed line represents the limit of detection. Data are depicted as medians for n = 8–10 individually analyzed mice. Statistical analyses were performed after log_10_-transformation using two-way ANOVA with Bonferroni’s post-hoc test. **(C)** Serum ALT levels 3 days post infection were determined. Levels higher than the dashed line are considered elevated and indicative of liver damage. Data are depicted as mean ± SEM for n = 5 individually analyzed mice. Statistical analyses were performed using two-tailed unpaired *t*-test with Welch’s correction (*p < 0.05, ***p < 0.001). **(D)** Female wild type and ADAPko mice were sacrificed 3 days post *Lm* infection. Pictures of the internal organs were taken to document the liver (▴) and spleen (▵) pathology. Three individual mice are shown for each genotype. **(E)** H&E staining of the spleens and livers 3 days post *Lm* infection. Organs were stored in 4% paraformaldehyde and later sectioned for histology and analyzed following H&E staining. Scale bars at the bottom right corner of each panel represent a distance of 200 µm for the spleen sections and 100 µm for the liver sections. **(F)** Scoring of degree of inflammation in the spleens and livers. Modified figure from (44). ADAP, adhesion and degranulation-promoting adaptor protein; ADAPko, ADAP knockout; CFU, colony-forming units; ALT, alanine aminotransferase.

To determine the overall consequences of ADAP-deficiency for the course of an *in vivo* infection, wild type and ADAPko mice were infected with *Lm* followed by comprehensive monitoring of morbidity, mortality, pathogen load (CFU), and immunopathology. Strikingly, ADAPko mice lost significantly more weight and exhibited higher lethality compared to wild type littermates (**Figure 1A**). Higher morbidity and mortality in ADAPko mice correlated with an early outgrowth of the pathogen and a significant delay in its elimination (**Figure 1B**). This was accompanied by higher serum alanine aminotransferase (ALT) levels in ADAPko mice (**Figure 1C**), indicative for a more severe liver damage in these animals. As depicted in **Figure 1D**, ADAPko mice showed serious and macroscopically visible signs of pathology in both organs, which was far more pronounced than in wild type animals. Moreover, histological examination of the spleen in wild type mice revealed that despite the white pulp showing areas of necrotic lesions, the basic structure of the white and red pulp remained generally intact on day 3 post *Lm* infection. In contrast, the base structure of spleens from *Lm*-infected ADAPko mice was largely destroyed, exhibited large areas of necrotic lesions, and showed more foci with leukocyte aggregates (**Figure 1E**). Consequently, ADAPko mice exhibited a higher inflammation score in both organs (**Figure 1F**). Taken together, ADAP-deficiency renders mice highly susceptible to infection with the intracellular pathogen *Lm*. Moreover, increased morbidity and mortality of ADAPko mice are associated with failures in pathogen control and enhanced immunopathology in the early phase of infection.

### Exaggerated Infiltration of Neutrophils and Monocytes Into Spleen and Liver of *Listeria monocytogenes*-Infected ADAPko Mice

**Figure 2:**
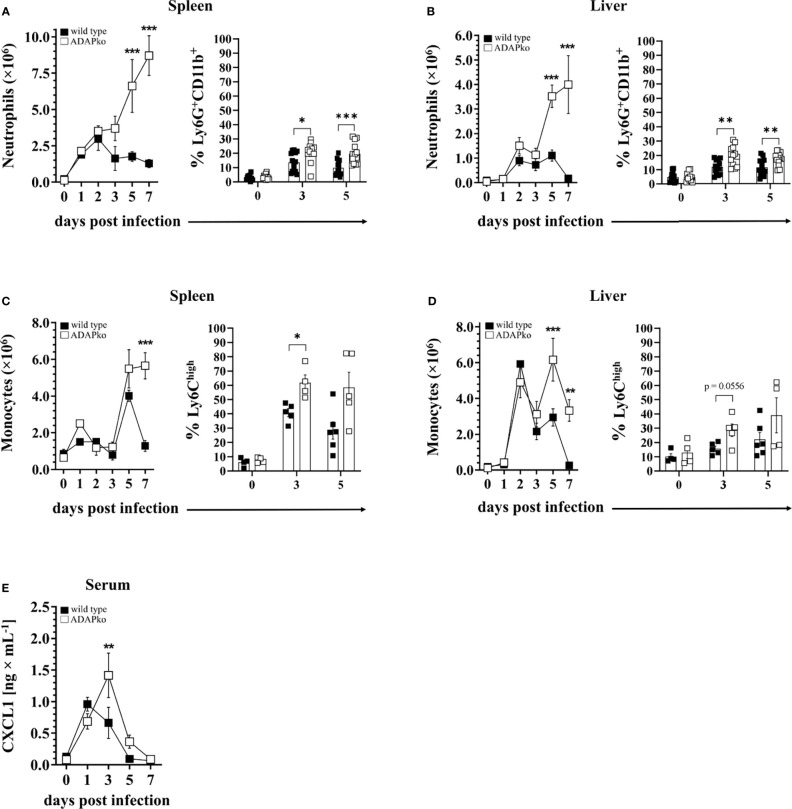
Excessive infiltration of neutrophils and monocytes into the spleen and liver of *Listeria monocytogenes (Lm)-*infected ADAPko mice. Wild type (▪) and ADAPko (▫) mice (age: 10–17 weeks) were either infected i.v. with 2.5 × 10^4^ CFU *Lm* (strain 10403S) or left untreated (uninfected control mice, day 0) and were sacrificed at the indicated times post infection. Leukocytes, gated as alive singlets and stained for CD45^+^Lin^−^, were isolated from the spleen and liver and subsequently **(A, B**, left panel**)** neutrophil (CD11b^+^Gr-1^+^Ly6G^+^ cells) and **(C, D**, left panel**)** monocyte (CD11b^+^Gr-1^+^Ly6G^−^ cells) absolute numbers as well as the frequencies of **(A, B**, right panel**)** neutrophils (CD11b^+^Ly6G^+^ cells) and **(C, D**, right panel**)** monocytes (CD11b^+^Ly6G^−^CX3CR1^low^Ly6C^high^ cells) were determined by flow cytometry. For all left panels, data are depicted as mean ± SEM for n = 4–5 individually analyzed mice, and statistical analyses were performed using two-way ANOVA with Bonferroni’s post-hoc test. Modified figure from (44). For all right panels, data shown are representative of three individual experiments **(A, B)** with n = 4–6 individually analyzed mice per group and one experiment **(C, D)** with n = 4–6 individually analyzed mice per group. **(E)** The concentrations of the neutrophil attracting chemokine CXCL1 was determined by cytometric bead array with n = 4 (uninfected control mice, day 0) and n = 8–9 (on the indicated times post infection) individually analyzed mice per group. Modified figure from (44). Data are depicted as mean ± SEM, and statistical analyses were performed using **(A–D)** unpaired, non-parametric Mann–Whitney test, and **(E)** analyses were performed using two-way ANOVA with Bonferroni’s post-hoc test (*p < 0.05, **p < 0.01, ***p < 0.001). ADAPko, adhesion and degranulation-promoting adaptor protein knockout; CFU, colony-forming units.

The pronounced leukocyte aggregates in *Lm*-infected ADAPko animals (**Figure 1E**) prompted us to have a closer look on the identity of leukocytes infiltrating the spleen and liver at different times post infection. Since enhanced weight loss and mortality of ADAPko mice develop between days 3 and 5 p.i., suggesting the protective innate immune response to be impaired in these mice (**Figure 1A**), we considered neutrophils and monocytes as primary targets for our analysis. Indeed, while the absolute numbers of neutrophils in the spleen of wild type mice peaked at 2 days p.i., the neutrophil numbers in the spleen of ADAPko mice continuously increased until the end of the survey, i.e., day 7 p.i. (**Figure 2A**, left panel). A similar pattern was observed for the frequencies of neutrophils in the spleen (**Figure 2A**, right panel) as well as for neutrophil numbers and frequencies in the liver (**Figure 2B**). Of note, prolonged neutrophil recruitment is well in line with increased levels of the neutrophil recruiting chemoattractant CXCL1 in the sera of *Lm*-infected ADAPko mice compared with their wild type counterparts (**Figure 2E**).

The dynamics of monocytes was found to be more complex. In the spleen, the absolute monocyte numbers remained largely constant early after *Lm* infection, before rapidly increasing on day 5 p.i., irrespective of the genotype (**Figure 2C**, left panel). Strikingly, the cell numbers drastically decreased again on day 7 in wild type animals, while they remained high in the spleen of ADAPko mice. Regarding the percentage, ADAPko mice showed significantly higher frequencies of Ly6C^high^ cells on day 3 and, in tendency, on day 5 post *Lm* infection (**Figure 2C**, right panel). Regardless of the genotype, the absolute monocyte number in the liver reached a first peak on day 2 and a second on day 5 p.i., with the latter being significantly higher in ADAPko mice (**Figure 2D**, left panel). Of note, while the monocyte numbers in the liver went back to normal levels in wild type mice on day 7 p.i., they remained high in ADAPko mice. No significant genotype-dependent differences were observed in the percentage of Ly6C^high^ monocytes in the liver following *Lm* infection (**Figure 2D**, right panel). Together, these finding show that during the early phase of *Lm* infection, exaggerated numbers of neutrophils and monocytes are recruited to the spleen and liver of ADAPko mice. However, despite their vast number, these phagocytes are inefficient in pathogen control early after infection.

### Increased Susceptibility to *Listeria monocytogenes* and Enhanced Immunopathology Following Infection in ADAPko Mice Cannot Be Directly Attributed to ADAP-Deficiency in Neutrophils and Monocytes

Since ADAPko mice were inefficiently controlling bacterial growth (**Figure 1B**) while at the same time the numbers of phagocytes were increased at sites of infection (**Figure 2**), we wondered whether ADAP-deficient phagocytes are functionally impaired, and as such, the observed phenotype could be attributed to ADAP-deficiency in phagocytes. Therefore, to prove this, we performed *Lm* infections in mice lacking ADAP specifically in phagocytes (ADAP^fl/fl^ × LysM-Cre^het^; conditional ADAPko).

Strikingly, lack of ADAP in phagocytes affected neither morbidity and mortality (**Supplementary Figure 1A**) nor early bacterial growth (CFU) in the spleen and liver of conditional ADAPko mice (**Supplementary Figure 1B**). In line with this, histological changes induced by *Lm* infection were independent of the genotype (**Supplementary Figures 1C, D**). Accordingly, conditional ADAPko and wild type mice showed similar and rather low serum ALT levels during the course of *Lm* infection, indicating only a marginal, genotype-independent liver damage (**Supplementary Figure 1**). Comparison of serum cytokine levels in *Lm*-infected conditional ADAPko mice and littermate controls revealed no alterations in the cytokine response in mice lacking ADAP in phagocytes with the exception of IFN-γ on day 3 p.i. (**Supplementary Figure 2**). In conclusion, ADAP-deficiency in phagocytes does not mirror the phenotype observed following *Lm* infection in conventional ADAPko mice.

### Infection-Associated Priming of Phagocytes in an ADAP-Deficient Host Induces an Altered Neutrophil and Monocyte Phenotype

**Figure 3:**
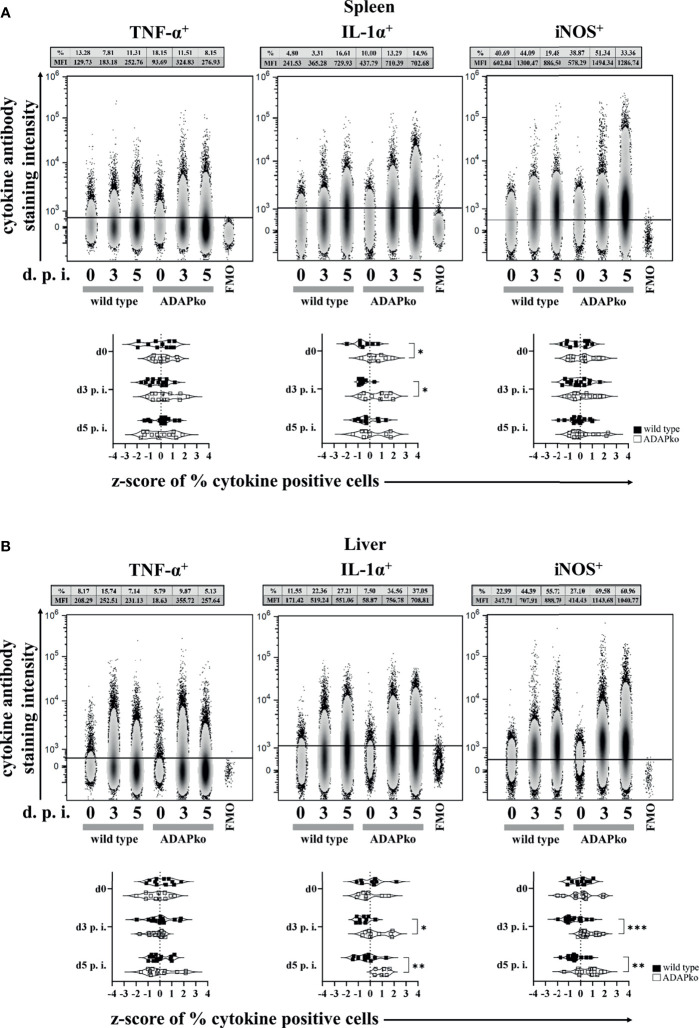
Neutrophils from ADAP-deficient mice show an altered inflammatory profile during *in vivo Listeria monocytogenes* (*Lm*) infection. Wild type (▪) and ADAPko (▫) mice (age: 10–17 weeks) were infected i.v. with 2.5 × 10^4^ CFU *Lm* (strain 10403S) or left untreated (uninfected control mice, day 0) and were sacrificed at the indicated times post infection. Leukocytes were isolated from **(A)** the spleen and **(B)** liver and were stimulated *in vitro* with PMA/ionomycin for 4 h After 1 h, Brefeldin A and Monensin were added. Cells were stained for Ly6G^+^CD11b^+^ cells in reference to CD45^+^Lin^−^ leukocytes. **(A, B)** Representative data (top panels) from one experiment with n = 4–6 individually analyzed mice per group were constrained to alive singlet Ly6G^+^CD11b^+^ neutrophils and are shown in columns side by side in a concatenated qualitative density plot (with outliers) in which each column represents data of all pooled mice from one genotype at a given time. Shown is the mean of cytokine positive neutrophils (in %) and the MFI (geometric mean) of all pooled mice per genotype in the top of the concatenated qualitative density plot. Summary plots (bottom panels) present the percentage of cytokine (TNF-α, IL-1α, and iNOS)-positive neutrophils, determined in reference to the corresponding FMO controls in a violin plot with all data points. Results from each independent experiment with n = 8–14 individually analyzed mice per group were normalized over all mice on a given day by z-score calculation. Resulting z-scores from two to three independent experiments are shown. Statistical analyses were performed using unpaired, non-parametric Mann–Whitney test (*p < 0.05, **p < 0.01, ***p < 0.001). ADAP, adhesion and degranulation-promoting adaptor protein; ADAPko, ADAP knockout; PMA, phorbol myristate acetate; MFI, mean fluorescence intensity; TNF, tumor necrosis factor; IL, interleukin; iNOS, inducible nitric oxide synthase; FMO, Fluorescence Minus One.

Puzzled by the striking discrepancy between exaggerated phagocyte accumulation in the spleen and liver of *Lm*-infected ADAPko mice (**Figure 2**) *versus* the strongly delayed pathogen elimination (**Figure 1B**), we next investigated whether infection-associated activation of phagocytes in an ADAP-deficient host would affect the production of the pro-inflammatory mediators TNF-α, IL-1α, and iNOS by these cells. While TNF-α production by neutrophils was not affected in the spleen (**Figure 3A**, left panels) and liver (**Figure 3B**, left panels), IL-1α production was significantly increased in *Lm*-infected ADAPko mice compared with wild type animals in the spleen (**Figure 3A**, middle panels) and liver (**Figure 3B**, middle panels). Of note, this difference was also observed in the spleen of naïve ADAPko mice. Moreover, hepatic neutrophils in ADAPko mice produced significantly more iNOS during the course of *Lm* infection (**Figure 3B**, right panels). Similar patterns were observed for inflammatory monocytes in the spleen and liver of ADAPko mice (**Supplementary Figure 3**). Noteworthy, in inflammatory monocytes, we also observed genotype-specific differences in TNF-α production (**Supplementary Figures 3A**, **3B**, left panels), and in contrast to neutrophils, inflammatory monocytes from both the spleen and liver of ADAPko mice produced significantly more iNOS following *Lm* infection (**Supplementary Figures 3A**, **3B**, right panels). Together, these data support the view that phagocytes primed during *Lm* infection within an ADAP-deficient environment develop a severely altered phenotype.

### ADAP and Tissue-Specific Alterations in the Gene Expression Profile of Neutrophils and Inflammatory Monocytes From Spleen and Liver of *Listeria monocytogenes*-Infected ADAPko Mice

**Figure 4:**
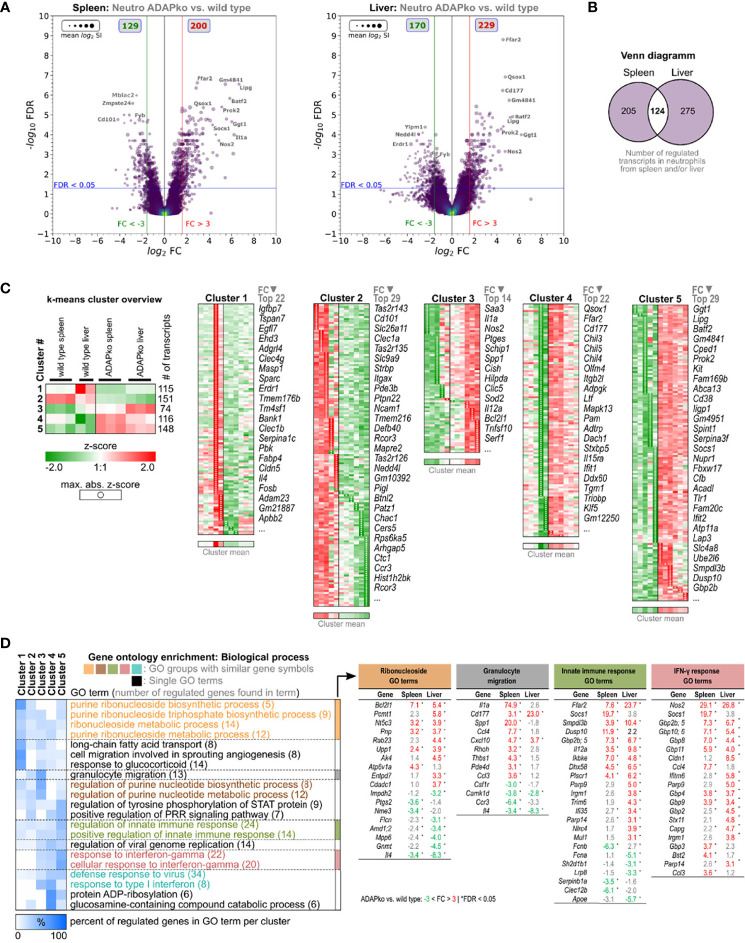
ǀ Microarray analysis of neutrophils from the spleen and liver of wild type and ADAPko mice 3 days post *in vivo Listeria monocytogenes* (*Lm*) infection. Wild type and ADAPko mice (age: 9–15 weeks) were infected i.v. with 2.5 × 10^4^ CFU *Lm* (strain 10403S) and sacrificed on day 3 post infection. Neutrophils (Ly6G^+^CD11b^+^ cells) were FACS-sorted from the spleen and liver. Per genotype and organ, neutrophils from six mice were isolated, and cells from two mice each were pooled, resulting in n = 2–3 independent replicate sample pools per organ and genotype. Total RNA was isolated and analyzed by Clariom S microarray (23 samples in total). Differentially expressed transcripts in spleen-/liver-derived neutrophils were determined comparing ADAPko *versus* wild type condition (fold change > ± 3-fold, FDR < 0.05). **(A)** Volcano plots of validly detected transcripts from indicated microarray comparisons, plotting −log_10_ FDR *versus* log_2_ fold change. Point sizes refer to mean log_2_ signal intensities (SIs) of transcripts calculated across all microarrays. Point color code reflects point density. FC (red/green vertical lines) and FDR (blue horizontal lines) criteria for differential gene expression are indicated. Gray boxes show numbers of significantly upregulated (red) and downregulated (green) transcripts. Gene symbols of selected transcripts are stated. **(B)** Venn diagram of 604 transcripts, differentially regulated in neutrophils isolated from the spleen or liver comparing ADAPko *versus* wild type condition, respectively. **(C)** Log_2_ SI data of the 604 transcripts were z-score transformed and k-means clustered (k = 5), and transcripts in resulting clusters were descendingly sorted by maximal absolute z-score (marked by black point indicators). Data represent color-coded z-scores. Stated gene symbols in each cluster are descendingly ranked by average absolute FC, showing the top n symbols. Top left subfigure: averaged representation of k-means clusters. Sample type, cluster ID, and numbers of genes per cluster are indicated. **(D)** Gene Ontology (GO) enrichment analysis for GO category “Biological Process” (FDR < 0.05, GO level ≥ 8). GO term groups with the highest significance and numbers of overrepresented genes for ADAPko *versus* wild type are shown. Data represent hierarchically clustered percentages of enriched genes (color code) that fall into indicated k-means clusters. Numbers in brackets represent number of enriched genes per GO term. ADAPko, adhesion and degranulation-promoting adaptor protein knockout; CFU, colony-forming units; FACS, fluorescence-activated cell sorting; FDR, false discovery rate; FC, fold change.

**Figure 5:**
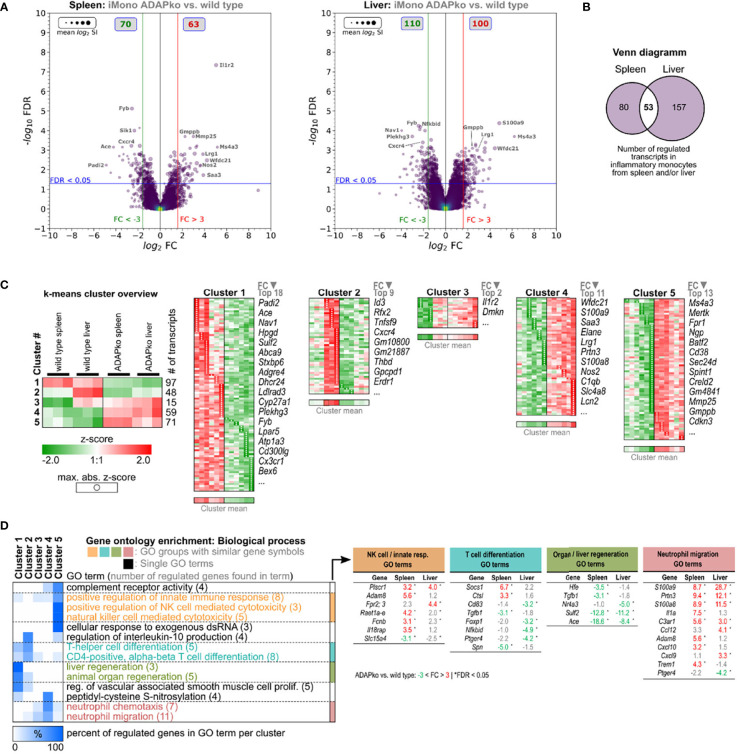
Microarray analysis of inflammatory monocytes from the spleen and liver of wild type and ADAPko mice 3 days post *in vivo Listeria monocytogenes* (*Lm*) infection. Wild type and ADAPko mice (age: 9–15 weeks) were infected i.v. with 2.5 × 10^4^ CFU *Lm* (strain 10403S) and sacrificed on day 3 post infection. Inflammatory monocytes (Ly6G^−^CD11b^+^ CX3CR1^low^Ly6C^high^ cells) were FACS-sorted from the spleen and liver. Per genotype and organ, inflammatory monocytes from six mice were isolated; and cells from two mice were pooled, resulting in n = 3 independent replicate sample pools per organ and genotype. Total RNA was isolated and analyzed by Clariom S microarray (23 samples in total). Differentially expressed transcripts in spleen-/liver-derived inflammatory monocytes were determined comparing ADAPko *versus* wild type condition (fold change > ± 3-fold, FDR < 0.05). **(A)** Volcano plots of validly detected transcripts from indicated microarray comparisons, plotting −log_10_ FDR *versus* log_2_ fold change. Point sizes refer to mean log_2_ signal intensities (SIs) of transcripts calculated across all microarrays. Point color code reflects point density. FC (red/green vertical lines) and FDR (blue horizontal lines) criteria for differential gene expression are indicated. Gray boxes show numbers of significantly upregulated (red) and downregulated (green) transcripts. Gene symbols of selected transcripts are stated. **(B)** Venn diagram of 604 transcripts, differentially regulated in inflammatory monocytes isolated from the spleen or liver comparing ADAPko *versus* wild type condition, respectively. **(C)** Log_2_ SI data of the 604 transcripts were z-score transformed and k-means clustered (k = 5), and transcripts in resulting clusters were descendingly sorted by maximal absolute z-score (marked by black point indicators). Data represent color-coded z-scores. Stated gene symbols in each cluster are descendingly ranked by average absolute FC, showing the top n symbols. Top left subfigure: averaged representation of k-means clusters. Sample type, cluster ID, and numbers of genes per cluster are indicated. **(D)** Gene Ontology (GO) enrichment analysis for GO category “Biological Process” (FDR < 0.05, GO level ≥ 8). GO term groups with the highest significance and numbers of overrepresented genes for ADAPko *versus* wild type are shown. Data represent hierarchically clustered percentages of enriched genes (color code) that fall into indicated k-means clusters. Numbers in brackets represent number of enriched genes per GO term. ADAPko, adhesion and degranulation-promoting adaptor protein knockout; CFU, colony-forming units; FACS, fluorescence-activated cell sorting; FDR, false discovery rate; FC, fold change.

Enhanced production of pro-inflammatory cytokines such as IL-1α and bactericidal mediators like iNOS by phagocytes is well in line with the observed increased immunopathology in ADAPko mice but is in contrast to the inefficient control of bacterial growth in mice lacking ADAP (**Figure 1B**). Thus, we performed unbiased *ex vivo* transcriptional profiling of phagocytes derived from *Lm*-infected mice to further characterize potential alterations in phagocyte phenotype and function on the gene expression level. To this end, we sorted neutrophils and inflammatory monocytes by FACS from total leukocyte suspensions of spleens and livers 3 days post *Lm* infection (**Figures 4**, **5**). Together, transcriptomes of 23 samples were assessed by Clariom S microarray analysis. For both cell subsets, lists of differentially expressed genes were compiled, comparing ADAPko *versus* wild type genotype and applying an FC (Fold change) cutoff of ±3-fold with FDR <0.05, as shown in volcano plots in **Figure 4A** for neutrophils and **Figure 5A** for inflammatory monocytes. As depicted in **Figure 4A**, comparing transcriptomes of spleen- and liver-derived neutrophils of ADAPko *versus* wild type mice on day 3 post *Lm* infection results in a plethora of differentially expressed genes. In general, we identified 200 upregulated and 129 downregulated transcripts in neutrophils from the spleen and 229 upregulated and 170 downregulated transcripts in liver-derived neutrophils. Thus, ADAP-deficiency in neutrophils seems to take effect in favor of gene-induction rather than transcriptional repression. Compared with neutrophils, transcriptional influence of ADAP-deficiency in inflammatory monocytes is less pronounced, with 70 (up in ADAPko) and 63 (down in ADAPko) transcripts in spleen-derived inflammatory monocytes, and 110 (up in ADAPko) and 100 (down in ADAPko) transcripts in their liver-derived counterparts (**Figure 5A**). Highly affected transcripts in neutrophils from both organs are, e.g., *Ffar2*, *Qsox1*, *Lipg*, *Batf2*, *Ggt1*, *Prok2*, and *Nos2*, all being significantly upregulated in the ADAPko genotype with some of them known for being functionally involved in neutrophil recruitment and induction by inflammatory conditions. Highly affected transcripts, similarly regulated in inflammatory monocytes from both organs, are e.g., *Cxcr4*, *Gmppb*, *Il1r2*, *Ms4a3*, *Nos2*, *S100a9*, and *Saa3*. As expected for the ADAPko genotype, expression of the ADAP encoding gene (*Fyb* transcript) is reduced compared with the wild type genotype in all comparisons for both cell subsets derived from both organs, thereby confirming ADAP-deficiency in comparison with wild type mice (FC in the spleen: neutrophils: −4.7, inflammatory monocytes: −5.9; FC in the liver: neutrophils: −2.9, inflammatory monocytes: −5.4).

To further compare organ-specific alterations induced by ADAP-deficiency side-by-side in more detail, lists of differentially regulated genes in cells from the spleen and liver were merged. This approach resulted in a total of 604 annotated transcripts differentially regulated in neutrophils and 290 transcripts generally regulated in inflammatory monocytes. Out of the 604 transcripts regulated in neutrophils, only 124 core transcripts were found to be regulated in neutrophils from both organs (**Figure 4B**). Interestingly, 205 transcripts were found to be regulated exclusively in spleen-derived neutrophils and 275 transcripts specifically in liver-derived neutrophils. Similarly, in inflammatory monocytes, out of 290 regulated transcripts, only 53 core transcripts showed differential expression in both the spleen and liver. Hence, 80 transcripts were specific for spleen-derived monocytes, and a majority of 157 transcripts were found to be specific for liver-derived inflammatory monocytes (**Figure 5B**), invoking the notion of tissue-specific *Lm*-induced transcriptional patterns within neutrophils and inflammatory monocytes in dependence of ADAP. In order to dissect expressional differences in ADAPko *versus* wild type neutrophils and inflammatory monocytes in more detail, normalized log_2_ signal intensities of the 604 and 290 identified transcripts, respectively, were z-score transformed and subjected to k-means cluster algorithm. Cluster numbering was based on averaged mean expression profiles of each cluster and ascendingly sorting them according to ADAPko conditions. GO enrichment analysis of clustered data for “Biological process” GO category is shown in **Figure 4D**. Here, ADAP-deficiency in neutrophils was found to be associated with GO terms like altered purine ribonucleoside/nucleotide metabolism, granulocyte migration, regulation of innate immunity, and response to IFN-γ sensing. Interestingly, IFN-γ response terms relate to several members of the family of guanylate-binding proteins (Gbp; *Gbp2*, *Gbp2b*, *Gbp3*, *Gbp4*, *Gbp5*, *Gbp6*, *Gbp8*, *Gbp10*, and *Gbp11*), known to function as IFN-γ-induced GTPases. They play a central role in protective immunity against microbial and viral pathogens (45) and are all significantly stronger expressed in ADAPko neutrophils, largely independent of the organ compartment. Since Gbps are induced upon IFN-γ-signaling, it is noteworthy that we indeed previously observed ADAPko mice to produce significantly elevated serum levels of IFN-γ on day 3 post *Lm* infection (38). Likely in consequence of the enhanced IFN-γ response, production of ROS in ADAPko neutrophils by the nitric oxide synthetase 2 (*Nos2*) seems to be favored, given *Nos2* FCs of 29.1 (spleen) and 26.8 (liver). However, there are also examples for transcriptionally repressed genes in ADAPko neutrophils. In cluster 2, *Cd101* (Immunoglobulin superfamily member 2), which together with Cxcr2 is used to differentiate Cxcr2^−^CD101^low^ immature from Cxcr2^+^CD101^+^ mature neutrophils (46), is the second most downregulated (FC spleen: −17.9, FC liver: −5.5) transcript (**Figure 4C**), thereby possibly indicating compromised maturity of ADAPko neutrophils.

The k-means clustering for the 290 differentially expressed genes from spleen- and liver-derived inflammatory monocytes of wild type and ADAPko mice on day 3 post *Lm* infection (**Figure 5C**) categorized them into groups of: 97 (cluster 1), 48 (cluster 2), 15 (cluster 3), 59 (cluster 4), and 71 (cluster 5) transcripts. Clustered data were used for GO enrichment analysis (FDR <0.05, GO level ≥8), and the results are shown in **Figure 5**. A closer look on GO term groups with the highest significance and numbers of overrepresented genes suggests ADAP-deficient inflammatory monocytes to have an altered phenotype regarding regulation of NK cell cytotoxicity, T cell differentiation, organ/liver regeneration, and neutrophil migration (**Figure 5D**). Especially, transcripts involved in neutrophil attraction/migration are consistently stronger expressed in ADAPko inflammatory monocytes. An interesting observation in splenic ADAPko inflammatory monocytes is the strong induction of neutrophil attractant *Il1*α (FC: 7.5) together with the upregulation of the IL-1α decoy receptor *Il1r2* (FC: 30), potentially antagonizing autocrine IL-1α sensing by ADAPko inflammatory monocytes themselves.

Despite the fact that the majority of regulated genes in neutrophils and inflammatory monocytes were regulated in either the spleen or liver, there are also common sense genes regulated in cells from both organs that we consider of special interest. Since ADAP is a receptor-associated signaling adaptor protein, we used a mouse receptor/ligand database (47) to filter the previously identified ADAP-dependent genes for known receptor/ligand interactions (**Supplementary Figure 4**). Receptor encoding common sense genes in neutrophils comprise *Adora2b*, *IL15r*α, *IL18bp*, *Tlr1*, and *Tmem67*. Common sense soluble ligands produced by neutrophils are *Cxcl10*, *Il4*, *Il12*α, *Il27*, *Prok2*, and *Spint1*, with only *Il4* showing reduced expression in neutrophils from ADAPko mice. Common sense receptors upregulated in ADAPko monocytes are *C3ar1*, *Fpr1*, *Il1r2*, and *Mertk*, whereas *Atp1a3*, *Cx3cr1*, *Cxcr4*, and *Plxnd1* are downregulated. Notably, these common sense receptors differ from those in neutrophils. In inflammatory monocytes from ADAPko mice, the only upregulated common sense ligands identified are *Lcn2*, *S100a8*, and *S100a9*. Altered expression of common sense receptors in ADAPko neutrophils and inflammatory monocytes render them interesting candidates for future analysis regarding involvement of ADAP in their associated signaling pathways or mechanisms responsible for their expression enhancement. Taken together, microarray analyses showed that neutrophils in general are transcriptionally more affected by ADAP-deficiency than inflammatory monocytes, regardless of their tissue origin. Moreover, it seems that observed ADAP-dependent transcriptional alterations in both cell subsets relate to the likewise ADAP-deficient tissue environment. This notion is based on the large extent of tissue-specific differential gene expression in neutrophils and inflammatory monocytes of splenic or hepatic origin.

### Reduced Phagocytic Capacity of *Listeria monocytogenes*-Primed Neutrophils in ADAP-Deficient Mice

**Figure 6:**
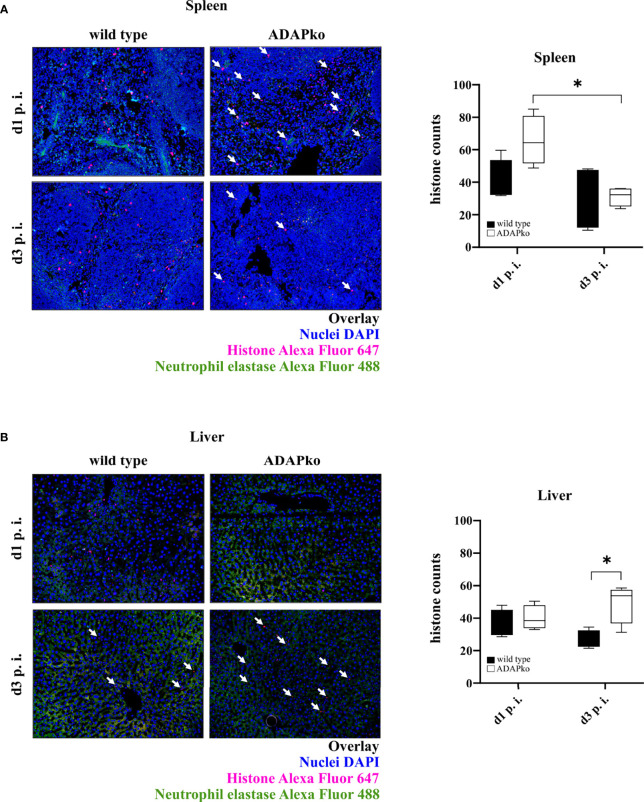
Distinct changes in NET formation by neutrophils from ADAP-deficient mice in response to *in vivo Listeria monocytogenes* (*Lm*) infection. Wild type (▪) and ADAPko (▫) mice (age: 10–14 weeks) were infected i.v. with 2.5 × 10^4^ CFU *Lm* (strain 10403S) and sacrificed at the indicated times post infection. Formalin-fixed tissue slices were stained with primary antibody (neutrophil elastase (NE) and histone H3) and subsequently with the secondary antibody (Alexa Fluor 488 donkey anti-rabbit IgG and goat anti-rat IgG Alexa Fluor 647) as well as DAPI for nuclei staining. Four pictures per slide (three different layers) at a magnification of ×200 were taken, and histones were counted using Image-Pro Plus 6 (double-blinded). Representative pictures show the overlay of the nuclei (colored in blue), histones (colored in red), and neutrophil elastase (colored in green) in **(A)** spleen and **(B)** liver tissues, whereas white arrows highlight the histones. Summary plots show the means of counted histones. Data are depicted as box and whiskers ± min to max for n = 4 individually analyzed mice per genotype out of one experiment. Statistical analyses were performed using two-way ANOVA with Bonferroni’s post-hoc test (*p < 0.05). NET, neutrophil extracellular trap; ADAP, adhesion and degranulation-promoting adaptor protein; ADAPko, adhesion and degranulation-promoting adaptor protein knockout.

To next test whether and how phagocyte function in ADAPko mice is affected, we performed functional assays and assessed the formation of NETs as well as the phagocytic capability of neutrophils. To quantify NET formation, we stained histones in *Lm*-infected spleen and liver tissues. As shown in **Figure 6A**, we detected no significant differences in the quantity of NETs in spleens of ADAPko animals compared with the wild type counterparts. Interestingly, while the number of detectable NETs in spleens of ADAPko mice was significantly reduced from day 1 to day 3 post *Lm* infection, this effect was not observed in wild type mice (**Figure 6A**, right panel). NET counts in livers of infected mice showed a different picture. While we did not observe differences in NET numbers on day 1 p.i., the number of NETs was significantly increased in ADAPko mice compared with wild type mice on day 3 p.i. (**Figure 6B**). Taken together, ADAP-deficiency results in minor but distinct changes in NET formation by neutrophils in *Lm*-infected organs.

**Figure 7:**
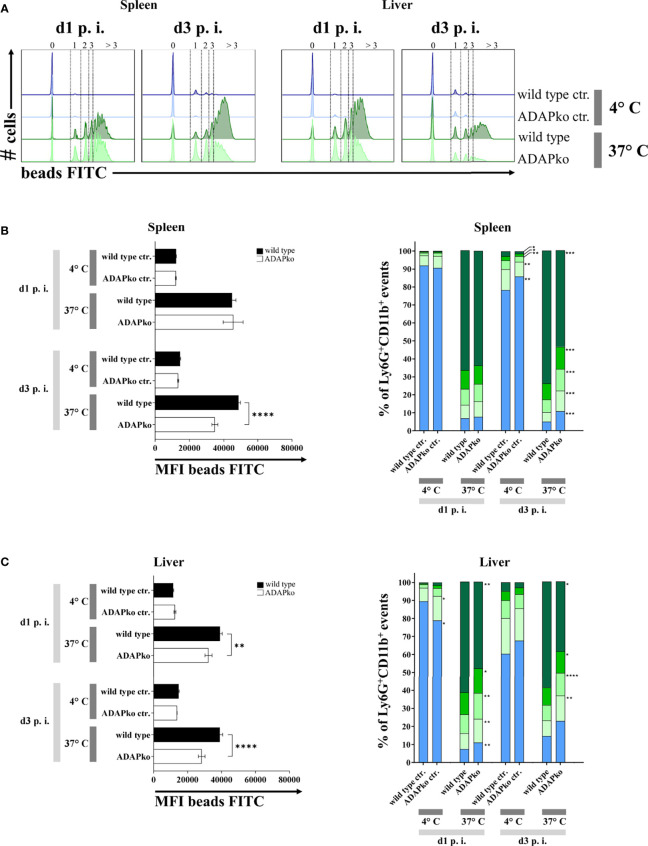
*In vivo Listeria monocytogenes*-primed neutrophils from ADAPko mice are functionally impaired in their phagocytic capacity. Wild type (▪) and ADAPko (▫) mice (age: 10–17 weeks) were infected i.v. with 2.5 × 10^4^ CFU *Lm* (strain 10403S) and sacrificed at the indicated times post infection. Leukocytes were isolated from the spleen and liver and stained for Ly6G^+^CD11b^+^ neutrophils in reference to CD45^+^Lin^−^ cells; and phagocytosis of Ly6G^+^CD11b^+^ cells was assessed by a 2 h incubation of the cells with carboxylate-modified FITC-fluorescent latex microspheres at 37°C or 4°C serving as negative controls (ctr.) with a cell to bead ratio of 1:5. **(A)** Representative histograms for the spleen (left histograms) and liver (right histograms) for Ly6G^+^CD11b^+^ neutrophils in wild type and ADAPko mice in addition to the related negative control 1 and 3 days post *Lm* infection. Numbers and dotted lines of the **(A)** histograms and the **(B**, **C**, right panels**)** bar charts (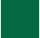 > 3 beads, 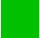 3 beads, 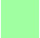 2 beads, 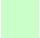 1 bead, and 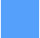 0 beads) show the fractioned cells according to the amount of incorporated beads. Phagocytic capability of **(B**, left panel**)** spleen and **(C**, left panel**)** liver neutrophils was considered by the MFI of bead-positive populations. Data are depicted as mean ± SEM for n = 6–8 individually analyzed mice per group out of two independent experiments. **(B**, **C**, left panels**)** Statistical analyses were performed using two-way ANOVA with Bonferroni’s post-hoc test and **(B**, **C**, right panels**)** two-tailed unpaired *t*-test with Welch’s correction (**p < 0.01, ****p < 0.0001). ADAPko, adhesion and degranulation-promoting adaptor protein knockout; CFU, colony-forming units; FITC, fluorescein isothiocyanate; MFI, mean fluorescence intensity. *p < 0.05, ***p < 0.001.

To further define potential functional alterations in phagocytes that might explain the inefficient pathogen control in ADAPko mice (**Figure 1B**), we had a closer look at the phagocytic activity of neutrophils and inflammatory monocytes from *Lm*-infected mice. To this end, we performed a FACS-based *ex vivo* phagocytosis assay using fluorescence-labeled latex microspheres. While on day 1 p.i. we did not observe any genotype-dependent differences in neutrophil phagocytosis in the spleen, a striking and highly significant decrease in the phagocytic capacity of neutrophils from ADAPko became evident by day 3 p.i. (**Figures 7A, B**, left panel). In the liver, the genotype-dependent difference was already detected on day 1 post *Lm* infection and became even more pronounced by day 3 (**Figures 7A, C**, left panel). Based on the intensity of the FITC-fluorescent signal, we could distinguish the cells according to the absolute number of incorporated beads, which were generally lower in ADAPko than in wild type mice (**Figures 7B, C**, right panels). Of note, for inflammatory monocytes, we did not observe genotype-specific effects on phagocytosis with the exception of day 1 p.i. in the liver, where inflammatory monocytes from ADAPko mice showed reduced phagocytic activity (**Supplementary Figure 5**). In conclusion, *ex vivo* phagocytosis assay uncovered functional impairment of neutrophils that were primed in the ADAP-deficient mice. This might explain at least in part the inability of ADAPko mice to limit bacterial growth and, in consequence, the fatal outcome of the infection.

## Discussion

We show here for the first time that ADAP-deficiency renders the host highly susceptible to infection with an intracellular pathogen and that impaired pathogen control is associated with profound molecular and functional alterations in phagocytes.

Strikingly, upon *Lm* infection, ADAPko mice show significantly enhanced pathology in the liver and spleen, i.e., major target organs of the pathogen (**Figure 1**). We consider a vicious cycle of excessive bacterial load, exaggerated accumulation of phagocytes and pro-inflammatory mediators released by these cells that in sum are indicative for a maladapted innate immune response to the pathogen as the underlying cause. Remarkably though, we do not observe these inflammatory changes in conditional knockout mice lacking ADAP exclusively in phagocytes (**Supplementary Figure 1** and **Supplementary Figure 2**). At a first glance, this hints at an extrinsic effect rather than an intrinsic effect of ADAP in phagocyte activation and effector function during *Lm* infection. However, at a closer view, it does not rule out the necessity for a combination of both aspects.

Regarding possible extrinsic effects of ADAP-deficiency on phagocyte function, we consider ADAP-dependent alterations in cytokine and chemokine expression by innate immune cells, in response to the infection, to play a key role in modulating the recruitment and inflammatory priming of neutrophils and inflammatory monocytes. Phagocytosis and killing of pathogens are, among others, induced by pro-inflammatory cytokines such as IFN-γ (48, 49) and TNF-α (50, 51). Additionally, next to neutrophils themselves (52), TNF-α is produced by macrophages and monocytes and stimulates NK cells to produce IFN-γ, which in turn increases the intracellular killing capacity of phagocytes. Potential failures in the TNF-α/IFN-γ regulatory network, and consequently reduced bactericidal capacity of macrophages and monocytes, could explain the increased bacterial loads in ADAP-deficient mice. However, while ADAP-deficient NK cells indeed produce lower levels of IFN-γ following *in vitro* stimulation, we did not observe any effect of ADAP-deficiency on IFN-γ production by NK cells primed during *in vivo Lm* infection (38). Moreover, we have previously shown that both TNF-α and IFN-γ levels are in fact significantly higher in ADAPko than in wild type mice following *Lm* infection (38), therefore excluding reduced abundance of these mediators as the underlying phagocyte-extrinsic mechanism for altered phagocyte priming.

By microarray analysis, we identified IL-15 receptor α (IL-15rα) to be consistently upregulated on splenic and hepatic neutrophils from infected ADAPko mice (**Supplementary Figure 4**). IL-15 promotes neutrophil migration, activation, and phagocytosis. Since ADAP is involved in the downstream signaling of IL-15rα, its overexpression in ADAP-deficient neutrophils might represent a cell-intrinsic mechanistic link to the reduced phagocytic capacity of ADAP-deficient neutrophils. The multi-subunit IL-15 receptor consists of the IL-15rα, IL-2/15 receptor β (CD122) and common cytokine receptor γ chain (Gammac or CD132) subunits, and is highly expressed on neutrophils (53). IL-15 receptor engagement in human neutrophils stimulates phagocytosis in dependency of the tyrosine-protein kinase Syk, which physically interacts with IL-15rα and becomes phosphorylated (54). Importantly, ADAP is known to interact with Syk and together with SLP-76 activates downstream Bruton’s tyrosine kinase (Btk) (10). Btk is considered a key kinase in many innate immunity signaling networks, as reviewed in (55). Blocking of IL-15rα was shown to reduce phagocytic capacity of human neutrophils by 40% (54). Of note, Coppolino et al. reported that among others, SLP-76 is needed for phagosome formation. Next to the initiation of phagocytosis, a molecular complex consisting of ADAP, SLP-76, and other molecules is formed, and the activation of Fcγ receptors leads to signaling events throughout phagocytosis that regulates actin polymerization indispensable for phagocytosis (9). Thus, ADAP-deficiency may cell-intrinsically prevent efficient enhancement of neutrophil phagocytosis by abrogating Btk activation, despite enhanced expression of IL-15rα on ADAPko neutrophils and solid levels of IL-15 in ADAPko sera (38). This hypothesis, of course, lacks experimental validation by future experiments.

The recruitment of neutrophils is required for initiation of an inflammatory response (56) but needs to be regulated to prevent excessive tissue damage (57). The observed exaggerated infiltration of neutrophils into the spleen and liver of ADAPko animals was unexpected (**Figures 2A, B**), since neutrophils deficient in ADAP itself or in ADAP interaction partners, such as SLP-76, were impaired in transmigration, rolling, and adhesion in mouse models of sterile inflammation. SLP-76 is an immune cell adaptor that is associated with phosphorylated ADAP *via* the Src kinase Fyn (4, 58). Hence, one might expect similar neutrophil phenotypes in ADAPko and SLP-76ko neutrophils. The latter were shown to have defects in integrin ligation, lacking activation of important integrin downstream regulators, and hence, they are unable to efficiently spread after direct β2-integrin stimulation, which provides compelling evidence for a critical role of SLP-76 in the manifestation of neutrophil attraction (59). Furthermore, in a sterile inflammation model of AKI, Block et al. revealed that neutrophils deficient in ADAP showed an impaired transmigration behavior into tissues (10). More specifically, they demonstrated that the interaction between SLP-76 and ADAP is necessary for the LFA-1 activation as well as for the recruitment of leukocytes into the inflamed tissue. Loss of ADAP resulted in an impaired rolling and adhesion to the endothelium and transmigration, leading to marked defects in which ADAPko mice were less susceptible to AKI (10). In contrast, systemic *Lm* infection and in consequence a pronounced tissue-specific innate immune activation appears to be sufficient to compensate or even overcompensate possible impairment in recruitment of ADAP-deficient neutrophils that have been observed in non-infectious experimental *in vivo* settings. The molecular basis of such potential phagocyte-intrinsic ADAP-dependent compensatory mechanism resulting in prolonged recruitment of ADAP-deficient neutrophils requires further investigations.

Next to TNF-α and IFN-γ, the IL-1α concentration was increased in sera of *Lm*-infected ADAPko mice (38). IL-1α can induce neutrophil recruitment (29), drives immunopathology, and is involved in several pathophysiological processes (60). Increased serum levels of IL-1α in combination with increased levels of the neutrophil recruiting chemoattractant CXCL1 (**Figure 2E**) might provide a conceivable phagocyte-extrinsic explanation for the massive neutrophil influx in the spleen and liver of ADAPko mice. However, recruited ADAP-deficient neutrophils appear to be inefficient in controlling pathogen growth, suggesting that they were not appropriately activated within the ADAP-deficient immune system. Of note, during *Lm* infection, monocytes are more efficient than neutrophils in the production of TNF-α and IL-1α (19), which is well in line with our data (**Figure 3** and **Supplementary Figure 3**, left and middle panels). Moreover, production of TNF-α might enhance the ability of neutrophils to take up and kill *Lm* (19). Our data, however, did not reveal any correlation between TNF-α production and antibacterial function of neutrophils.

Neutrophils can kill bacteria by release of NETs containing histones as powerful antimicrobials. This NETosis-termed cell death requires the formation of ROS, which can cause tissue damage (61) but is bactericidal on its own. Several agonists trigger NET formation, including cytokines, microbial components, and bacteria itself. During formation of NETs, histones are released into the surrounding tissues, which are toxic for the invading pathogens but can also cause tissue damage (62). Our data suggest that in the spleen of *Lm*-infected mice, neutrophils switch from NETosis on day 1 p.i. to phagocytosis on day 3, which is supported by the significantly reduced number of histones released from neutrophils in ADAPko mice (**Figure 6A**) and at the same time increased phagocytosis (**Figures 7A, B**) on day 3 post infection. Such a functional switch has indeed been described before (63, 64) and is well in line with our findings in the liver on day 3 post *Lm* infection. Here, neutrophils from ADAPko mice generated more histones than their wild type. counterparts (**Figure 6B**), while in direct contrast, the phagocytic capacity of ADAPko neutrophils was reduced (**Figures 7A, C**). As we did not observe any obvious failure of NET release in ADAPko mice, it remains elusive if and to which extent the observed distinct differences in NET formation would affect pathogen control in these mice.

Our transcriptome data integrate aspects of both phagocyte-intrinsic effects and phagocyte-extrinsic effects resulting from altered tissue environmental imprinting in the ADAP-deficient host. They clearly indicate that pro-inflammatory pathways are preferentially switched on in both, infection-primed neutrophils and monocytes from ADAPko mice, while at the same time, cellular processes contributing to tissue regeneration are less active, especially in monocytes (**Figures 4**, **5**). Collateral tissue damage is known to result from secreted proteases and microbicidal substances at sites of infection, such as ROS, produced by neutrophils, as well as monocytes (65). In addition to ROS, neutrophils can generate reactive nitrogen species through the expression of iNOS, an enzyme that converts l-arginine into nitric oxide (NO^−^). Mice deficient in the iNOS encoding gene *Nos2* show increased susceptibility to bacterial infection (20). Of note, enhanced expression of *Il1α* and *Nos2*, as identified by flow cytometry on the protein level in neutrophils and inflammatory monocytes in *Lm*-infected ADAPko mice (**Figure 3** and **Supplementary Figure 3**), is well in line with the microarray data (**Figures 4**, **5**). Thus, it appears that phagocytes from ADAPko mice are drivers of inflammation, which is supported by the pronounced immunopathology in the spleen and liver of *Lm*-infected ADAPko animals.

An important phagocyte-extrinsic yet still ADAP-dependent factor with potential impact on the observed neutrophil and monocyte phenotypes might be the production of anti-inflammatory TGF-β. Interestingly, we found the TGF-β plasma levels in ADAPko to be reduced already in naïve mice. Moreover, increasement of TGF-β was delayed in ADAP-deficient animals early on during *Lm* infection (**Supplementary Figure 6**). Of note, platelets might be mechanistically involved in this abnormal response, since they store high amounts of TGF-β (66). We have shown before that the platelet-specific deletion of ADAP augments experimental autoimmune encephalomyelitis (EAE) in mice, suggesting a regulatory role for ADAP in platelets during EAE (33, 67). Hence, impaired TGF-β production in ADAP-deficient mice might partially relate to the observed pro-inflammatory transcriptional profile of ADAP-deficient neutrophils and monocytes.

While ultimately we cannot conclude from our data, which of the observed effects on phagocyte phenotype and functionality most prominently affects the outcome of listeriosis in ADAPko, we are puzzled by the clear dichotomy between phagocyte accumulations at sites of infection and inefficient pathogen control (**Figures 1**, **2**). We consider that impaired early control of *Lm* in ADAPko mice and with this, uncontrolled bacterial growth results in prolonged attraction of phagocytes that in turn induce severe immunopathology in the spleen and liver of these mice (68). As can be expected for a multifaceted signaling adaptor protein like ADAP, consequences of its deficiency in neutrophils and inflammatory monocytes can only be interpreted in context of relevant inflammatory signaling events that the cells are exposed to in a tissue-specific manner. In this light and given the ameliorated disease severity in phagocyte-specific conditional ADAPko, it remains difficult to distinguish cell-intrinsic and tissue-environmental consequences of ADAP-deficiency in the conventional ADAPko mouse model. Nevertheless, our analyses give new clues as to which signaling pathways within neutrophils and inflammatory monocytes may depend on ADAP as a signaling adaptor in a naturally orchestrated, innate-driven *in vivo* infection.

## Data Availability Statement

The original contributions presented in the study are publicly available. These data can be found here: https://www.ncbi.nlm.nih.gov/geo/query/acc.cgi?acc=GSE175993.

## Ethics Statement

The animal study was reviewed and approved by Niedersächsisches Landesamt für Verbraucherschutz und Lebensmittelsicherheit (33.9.42502-04-10/0256) as well as the local government agencies (Landesverwaltungsamt Sachsen-Anhalt; AZ 42502-2-1603 UniMD).

## Author Contributions

MB designed the experiments, performed the experiments, analyzed the data, and wrote the manuscript. MB, GP, AJ, and HD designed and performed the experiments and visualized the data. MB and AJ performed statistical analysis and visualized the microarray data. OK and BX performed experiments and visualized data. BR, DS, and ID contributed conception to the analysis and data interpretation and provided research material. AR, BS, and DB planned, designed, and supervised the research and wrote the manuscript. All authors contributed to the article and approved the submitted version.

## Funding

This research was funded by the Deutsche Forschungsgemeinschaft (DFG, German Research Foundation)—Project-ID 97850925-SFB 854 (BS B19, DB A23). MB and GP were supported by grants (SI2 and SI3) of the State of Saxony-Anhalt to BS.

## Conflict of Interest

The authors declare that the research was conducted in the absence of any commercial or financial relationships that could be construed as a potential conflict of interest.

## Publisher’s Note

All claims expressed in this article are solely those of the authors and do not necessarily represent those of their affiliated organizations, or those of the publisher, the editors and the reviewers. Any product that may be evaluated in this article, or claim that may be made by its manufacturer, is not guaranteed or endorsed by the publisher.

## Supplementary Material

Supplementary Figure 1 Disease phenotype of conditional ADAPko mice resembles that of *Listeria monocytogenes*-infected wild type mice. ADAP^fl/fl^ × LysM-Cre^het^ (▫) mice (age: 9-14 weeks) lacking ADAP specifically in phagocytes and respective littermate controls (ADAP^wt/wt^ × LysM-Cre^het^, ▪) were infected i. v. with **(A)** 1 × 10^5^ CFU and **(B–E)** 2.5 × 10^4^ CFU *Lm* (strain 10403S) or left untreated (uninfected control mice, day 0) and were sacrificed at the indicated times post infection. **(A)** Infected mice were weighed and monitored daily and the survival was reported. Data are depicted as mean ± SEM for n = 8 individually analyzed mice per group out of two independent experiments. Statistical analyses were performed using two-way ANOVA with Bonferroni’s *post hoc* test for body weight data and Mantel-Cox log-rank test for the survival data. **(B)** Bacterial loads in spleen and liver after *Lm* infection. The dashed line represents the limit of detection. Data are depicted as medians for n = 3-6 individually analyzed mice. Statistical analyses were performed after log_10_-transformation using two-way ANOVA with Bonferroni’s *post hoc* test. **(C)** H&E staining of spleens and livers 3 days post infection. Organs were stored in 4 ml 4% paraformaldehyde and later sectioned for histology and analyzed following H&E staining. Histological analyses of spleen and liver revealed acute, necrotizing inflammatory changes in both organs. In the spleen, the follicular centers were particularly more affected, whereas the liver showed multifocal, acute and randomly distributed, necrotizing hepatitis. Scale bars at the bottom right corner of each panel represent a distance of 100 µm for the spleen sections and 200 µm as well as 50 µm for the liver sections. **(D)** Scoring of degree of inflammation in spleens and livers. Data are depicted for n = 2-5 individually analyzed mice. **(E)** Serum ALT levels were determined. Levels higher than the dashed line are considered elevated and indicative of liver damage. Data are depicted as mean ± SEM for n = 3-13 individually analyzed mice per group out of at least two independent experiments. Statistical analyses were performed using two-tailed unpaired *t*-test with Welch’s correction.

Supplementary Figure 2 Conditional ADAPko and wild type mice exhibit a similar serum cytokine profile following *Listeria monocytogenes* infection. ADAP^fl/fl^ × LysM-Cre^het^ (▫) mice (age: 9-16 weeks) lacking ADAP specifically in phagocytes and respective littermate controls (ADAP^wt/wt^ × LysM-Cre^het^, ▪) were infected i. v. with 2.5 × 10^4^ CFU *Lm* (strain 10403S) or left untreated (uninfected control mice, day 0) and were sacrificed at the indicated times post infection. Cytokine concentration (pg/ml) in serum of *Lm-*infected mice was analyzed *via* a flow cytometry based pre-defined multiplex detection assay. Summary plots present the average concentration of the analytes calculated from two replicates. Data are depicted as mean ± SEM for n = 5-10 individually analyzed mice per group out of at least two independent experiments. Horizontal dotted lines show the limit of detection. Protein concentrations under the detection limit were considered undetectable and hence were not included into statistics; numbers represent the detectable samples out of the total number of samples. If not otherwise stated all samples were detectable. Statistical analyses were performed using two-tailed unpaired *t*-test with Welch’s correction (*p < 0.05). n. d.; not detectable.

Supplementary Figure 3 Inflammatory monocytes derived from ADAP-deficient mice exhibit an altered inflammatory profile during *in vivo Listeria monocytogenes* infection. Wild type (▪) and ADAPko (▫) mice (age: 10-17 weeks) were infected i. v. with 2.5 × 10^4^ CFU *Lm* (strain 10403S) or left untreated (uninfected control mice, day 0) and were sacrificed at the indicated times post infection. Leukocytes were isolated from **(A)** spleen and **(B)** liver and were stimulated *in vitro* with PMA/ionomycin for 4 h. After 1 h Brefeldin A and Monensin were added. Cells were stained for CX3CR1^low^Ly6C^high^ cells in reference to CD45^+^Lin^-^Ly6G^-^CD11b^+^ leukocytes. **(A, B)** Representative data (top panels) from one experiment with n = 4-6 individually analyzed mice per group were constrained to alive singlet CX3CR1^low^Ly6C^high^ inflammatory monocytes and are shown in columns side-by-side in a concatenated qualitative density plot (with outliers) in which each column represents data of all pooled mice from one genotype at a given time. Shown is the mean of cytokine positive inflammatory monocytes (in %) and the MFI (geometric mean) of all pooled mice per genotype in the top of the concatenated qualitative density plot. Summary plots (bottom panels) present the percentage of cytokine (TNF-α, IL-1α and iNOS)-positive inflammatory monocytes, determined in reference to the corresponding FMO controls in a violin plot with all data points. Results from each independent experiment with n = 8-14 individually analyzed mice per group were normalized over all mice on a given day by z-score calculation. Resulting z-scores from 2-3 independent experiments are shown. Statistical analyses were performed using unpaired, nonparametric Mann-Whitney test (*p < 0.05, **p < 0.01, ***p < 0.001).

Supplementary Figure 4 Receptors and ligands differentially expressed in neutrophils and inflammatory monocytes from spleen and liver of *Listeria monocytogenes* infected ADAPko mice 3 days post infection. Gene symbols of differentially expressed transcripts from microarray analysis in and were filtered by a mouse receptor/ligand interaction database according to reference (47) and resulting gene lists were sorted by fold change. Differentially regulated (up: red, down: green) common sense genes found in cells isolated from spleen as well as liver are shown in bold letters and are indicated with an arrow.

Supplementary Figure 5 ǀ Phagocytic capacity of inflammatory monocytes following *in vivo Listeria monocytogenes* infection is largely independent of ADAP. Wild type (▪) and ADAPko (▫) mice (age: 10-17 weeks) were infected i. v. with 2.5 × 10^4^ CFU *Lm* (strain 10403S) and sacrificed at the indicated times post infection. Leukocytes were isolated from spleen and liver, stained for CX3CR1^low^Ly6C^high^ inflammatory monocytes in reference to CD45^+^Lin^-^Ly6G^-^CD11b^+^ cells and phagocytosis of CX3CR1^low^Ly6C^high^ cells was assessed by a 2 h incubation of the cells with carboxylate-modified FITC-fluorescent latex microspheres at 37°C or 4°C serving as negative controls (ctr.) with a cell to bead ratio of 1:5. **(A)** Representative histograms for spleen (left histograms) and liver (right histograms) for CX3CR1^low^Ly6C^high^ inflammatory monocytes in wild type and ADAPko mice in addition to the related negative control 1 and 3 days post *Lm* infection. Numbers and dotted lines of the **(A)** histograms and the (**B, C**, right panels) bar charts ( > 3 beads,  3 beads,  2 beads,  1 bead,  0 beads) show the fractioned cells according to the amount of incorporated beads. Phagocytic capability of (**B**, left panel) spleen and (**C**, left panel) liver inflammatory monocytes was considered by the MFI of bead-positive populations. Data are depicted as mean ± SEM for n = 6-8 individually analyzed mice per group out of two independent experiments. (**B**, **C**, left panels) Statistical analyses were performed using two-way ANOVA with Bonferroni’s *post hoc* test and (**B**, **C**, right panels) two-tailed unpaired *t*-test with Welch’s correction (**p < 0.01, ****p < 0.0001).

Supplementary Figure 6 ǀ TGF-β serum concentration of *Listeria monocytogenes* infected wild type and ADAPko mice. Cardiac blood was taken and serum samples were prepared. Serum concentration of TGF-β was determined by ELISA with n = 8-9 individually analyzed mice per group. Analysis for the frequency of TGF-β serum-positive animals was performed using Fisher’s exact test (*p < 0.05).

Supplementary Figure 7 ǀ FACS gating for neutrophils and inflammatory monocytes of wild type and ADAPko mice during *Listeria monocytogenes* infection. Representative gating scheme to define **(A)** neutrophils and **(B)** inflammatory monocytes from untreated (uninfected control mice, day 0) or *Lm*-infected (i. v. with 2.5 × 10^4^ CFU *Lm*) wild type and ADAPko mice at the indicated times post infection. Cells were pre-gated on leukocytes by means of FSC/SSC and doublets were excluded by FSC-A/FSC-H gating. Dead cells were eliminated by staining with live/dead marker and only CD45^+^ as well as lineage negative cells were further characterized (pre-gating not shown). Neutrophils were gated as Ly6G^+^CD11b^+^ cells and inflammatory monocytes as Ly6C^+^CX3CR1^high^.
