# Supplementary figures and images for: Enhanced Susceptibility of ADAP-Deficient Mice to Listeria monocytogenes Infection Is Associated With an Altered Phagocyte Phenotype and Function
Source: Front Immunol. 2021 Sep 30;12:724855. doi: 10.3389/fimmu.2021.724855 (PMC8515145; doi:10.3389/fimmu.2021.724855)

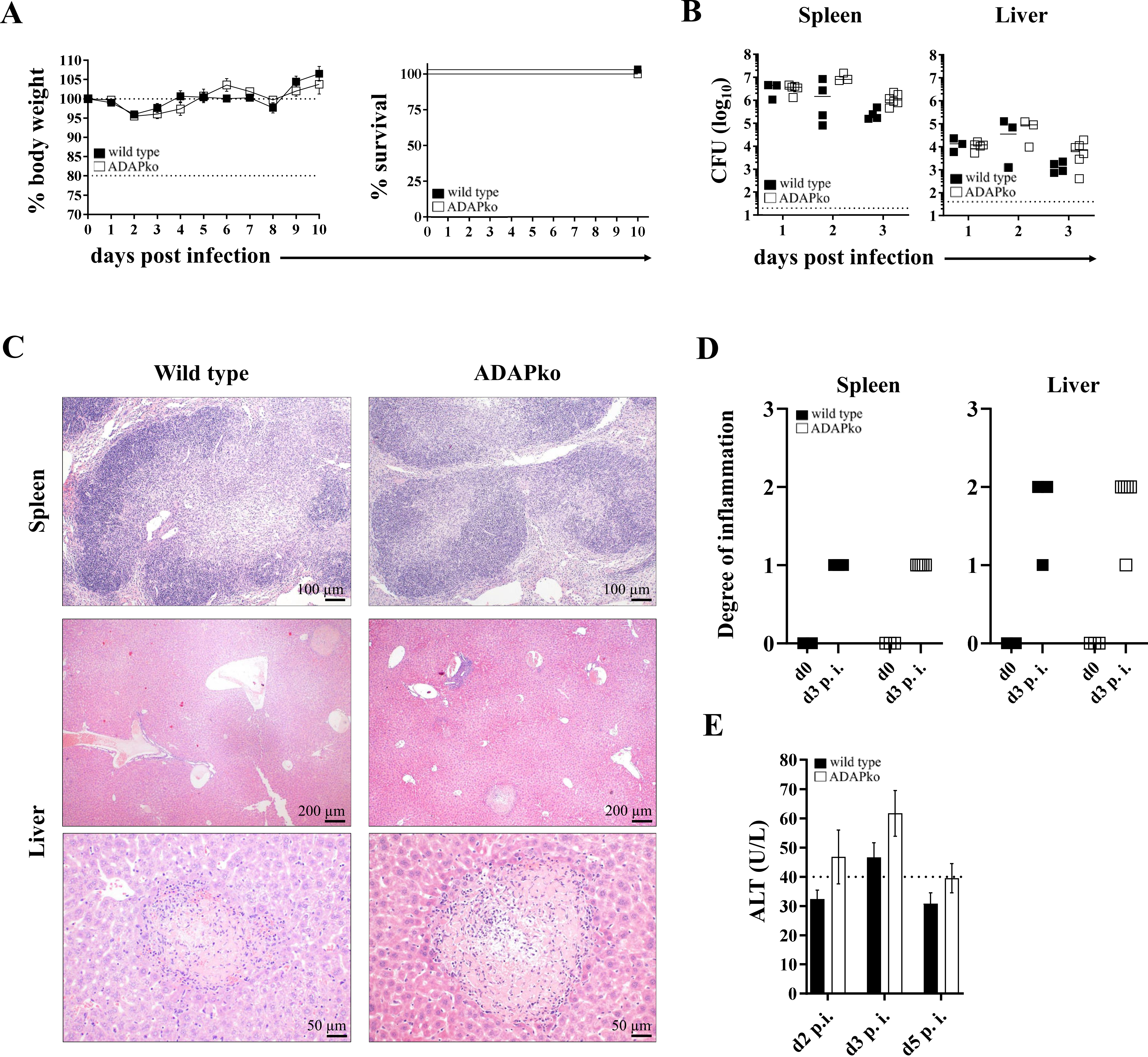

Supplement: Supplementary Figure 1 — Disease phenotype of conditional ADAPko mice resembles that of Listeria monocytogenes-infected wild type mice. ADAPfl/fl × LysM-Crehet (▫) mice (age: 9-14 weeks) lacking ADAP specifically in phagocytes and respective littermate controls (ADAPwt/wt × LysM-Crehet, ▪) were infected i. v. with (A) 1 × 10^5^ CFU and (B–E) 2.5 × 10^4^ CFU Lm (strain 10403S) or left untreated (uninfected control mice, day 0) and were sacrificed at the indicated times post infection. (A) Infected mice were weighed and monitored daily and the survival was reported. Data are depicted as mean ± SEM for n = 8 individually analyzed mice per group out of two independent experiments. Statistical analyses were performed using two-way ANOVA with Bonferroni’s post hoc test for body weight data and Mantel-Cox log-rank test for the survival data. (B) Bacterial loads in spleen and liver after Lm infection. The dashed line represents the limit of detection. Data are depicted as medians for n = 3-6 individually analyzed mice. Statistical analyses were performed after log10-transformation using two-way ANOVA with Bonferroni’s post hoc test. (C) H&E staining of spleens and livers 3 days post infection. Organs were stored in 4 ml 4% paraformaldehyde and later sectioned for histology and analyzed following H&E staining. Histological analyses of spleen and liver revealed acute, necrotizing inflammatory changes in both organs. In the spleen, the follicular centers were particularly more affected, whereas the liver showed multifocal, acute and randomly distributed, necrotizing hepatitis. Scale bars at the bottom right corner of each panel represent a distance of 100 µm for the spleen sections and 200 µm as well as 50 µm for the liver sections. (D) Scoring of degree of inflammation in spleens and livers. Data are depicted for n = 2-5 individually analyzed mice. (E) Serum ALT levels were determined. Levels higher than the dashed line are considered elevated and indicative of liver damage. Data are depicted as mean ± S [file Image_1.tiff]

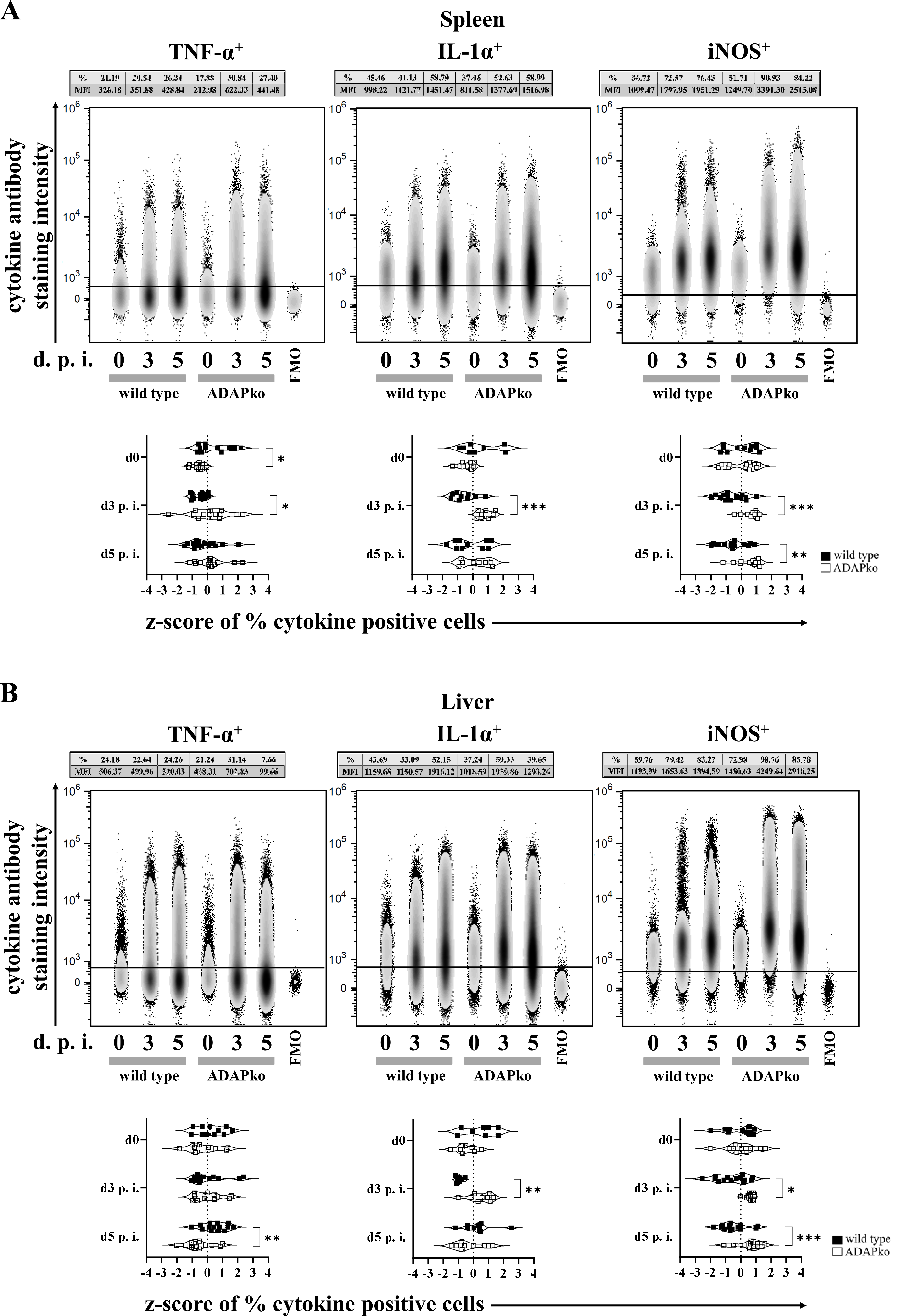

Supplement: Supplementary Figure 3 — Inflammatory monocytes derived from ADAP-deficient mice exhibit an altered inflammatory profile during in vivo Listeria monocytogenes infection. Wild type (▪) and ADAPko (▫) mice (age: 10-17 weeks) were infected i. v. with 2.5 × 10^4^ CFU Lm (strain 10403S) or left untreated (uninfected control mice, day 0) and were sacrificed at the indicated times post infection. Leukocytes were isolated from (A) spleen and (B) liver and were stimulated in vitro with PMA/ionomycin for 4 h. After 1 h Brefeldin A and Monensin were added. Cells were stained for CX3CR1lowLy6Chigh cells in reference to CD45+Lin-Ly6G-CD11b+ leukocytes. (A, B) Representative data (top panels) from one experiment with n = 4-6 individually analyzed mice per group were constrained to alive singlet CX3CR1lowLy6Chigh inflammatory monocytes and are shown in columns side-by-side in a concatenated qualitative density plot (with outliers) in which each column represents data of all pooled mice from one genotype at a given time. Shown is the mean of cytokine positive inflammatory monocytes (in %) and the MFI (geometric mean) of all pooled mice per genotype in the top of the concatenated qualitative density plot. Summary plots (bottom panels) present the percentage of cytokine (TNF-α, IL-1α and iNOS)-positive inflammatory monocytes, determined in reference to the corresponding FMO controls in a violin plot with all data points. Results from each independent experiment with n = 8-14 individually analyzed mice per group were normalized over all mice on a given day by z-score calculation. Resulting z-scores from 2-3 independent experiments are shown. Statistical analyses were performed using unpaired, nonparametric Mann-Whitney test (*p < 0.05, **p < 0.01, ***p < 0.001). [file Image_3.tiff]

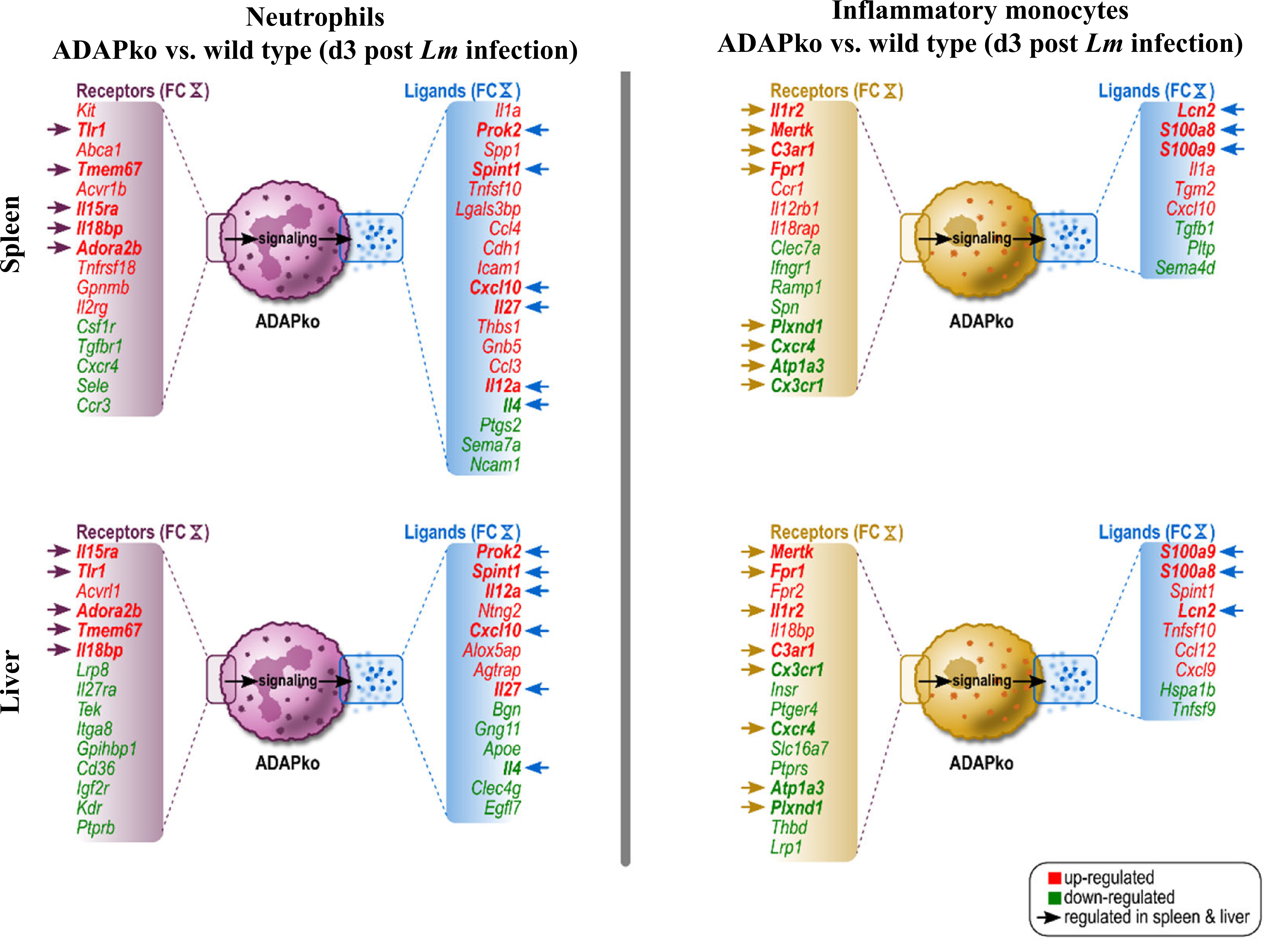

Supplement: Supplementary Figure 4 — Receptors and ligands differentially expressed in neutrophils and inflammatory monocytes from spleen and liver of Listeria monocytogenes infected ADAPko mice 3 days post infection. Gene symbols of differentially expressed transcripts from microarray analysis in and were filtered by a mouse receptor/ligand interaction database according to reference (47) and resulting gene lists were sorted by fold change. Differentially regulated (up: red, down: green) common sense genes found in cells isolated from spleen as well as liver are shown in bold letters and are indicated with an arrow. [file Image_4.tiff]

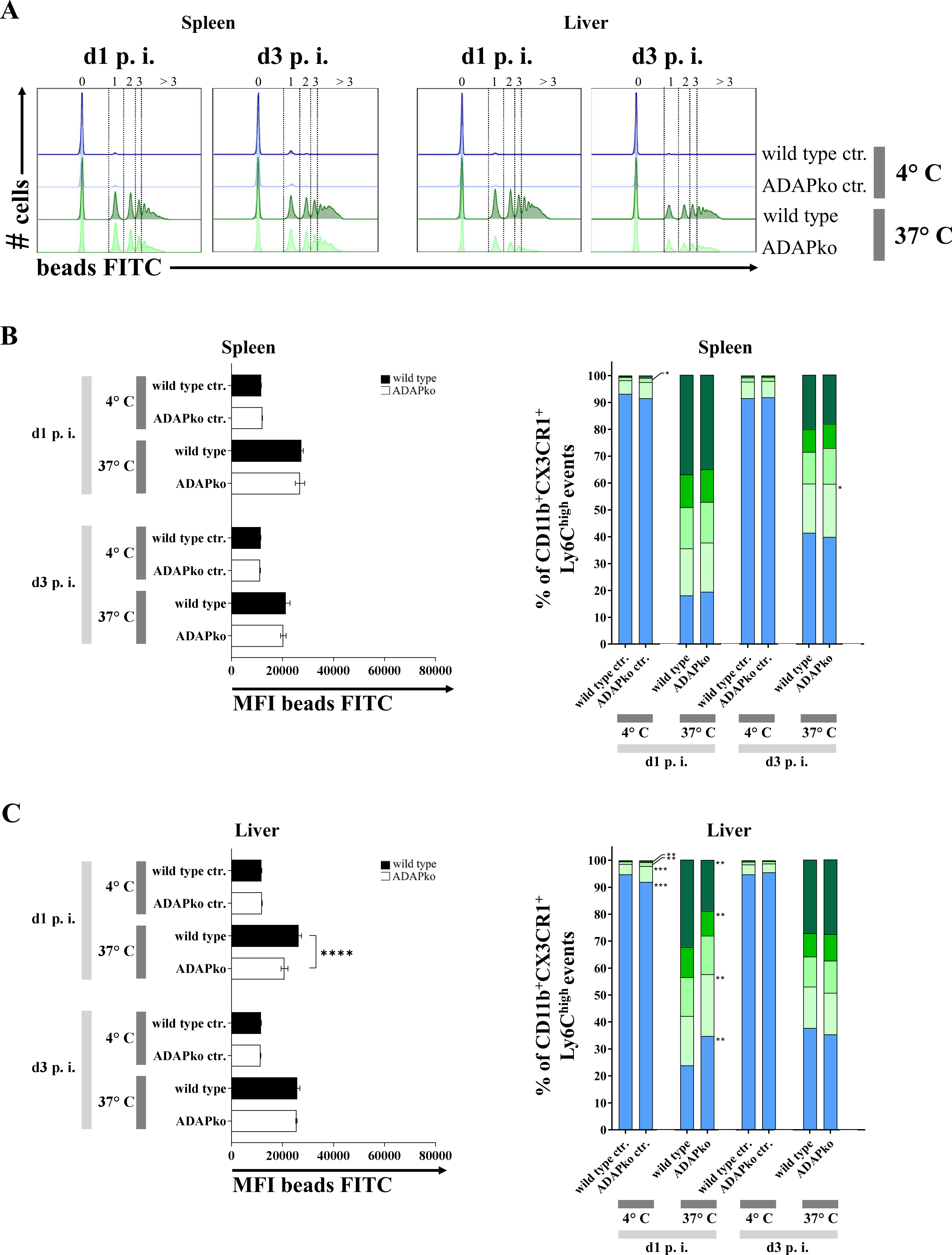

Supplement: Supplementary Figure 5 — ǀ Phagocytic capacity of inflammatory monocytes following in vivo Listeria monocytogenes infection is largely independent of ADAP. Wild type (▪) and ADAPko (▫) mice (age: 10-17 weeks) were infected i. v. with 2.5 × 10^4^ CFU Lm (strain 10403S) and sacrificed at the indicated times post infection. Leukocytes were isolated from spleen and liver, stained for CX3CR1lowLy6Chigh inflammatory monocytes in reference to CD45+Lin-Ly6G-CD11b+ cells and phagocytosis of CX3CR1lowLy6Chigh cells was assessed by a 2 h incubation of the cells with carboxylate-modified FITC-fluorescent latex microspheres at 37°C or 4°C serving as negative controls (ctr.) with a cell to bead ratio of 1:5. (A) Representative histograms for spleen (left histograms) and liver (right histograms) for CX3CR1lowLy6Chigh inflammatory monocytes in wild type and ADAPko mice in addition to the related negative control 1 and 3 days post Lm infection. Numbers and dotted lines of the (A) histograms and the (B, C, right panels) bar charts ( > 3 beads, 3 beads, 2 beads, 1 bead, 0 beads) show the fractioned cells according to the amount of incorporated beads. Phagocytic capability of (B, left panel) spleen and (C, left panel) liver inflammatory monocytes was considered by the MFI of bead-positive populations. Data are depicted as mean ± SEM for n = 6-8 individually analyzed mice per group out of two independent experiments. (B, C, left panels) Statistical analyses were performed using two-way ANOVA with Bonferroni’s post hoc test and (B, C, right panels) two-tailed unpaired t-test with Welch’s correction (**p < 0.01, ****p < 0.0001). [file Image_5.tiff]

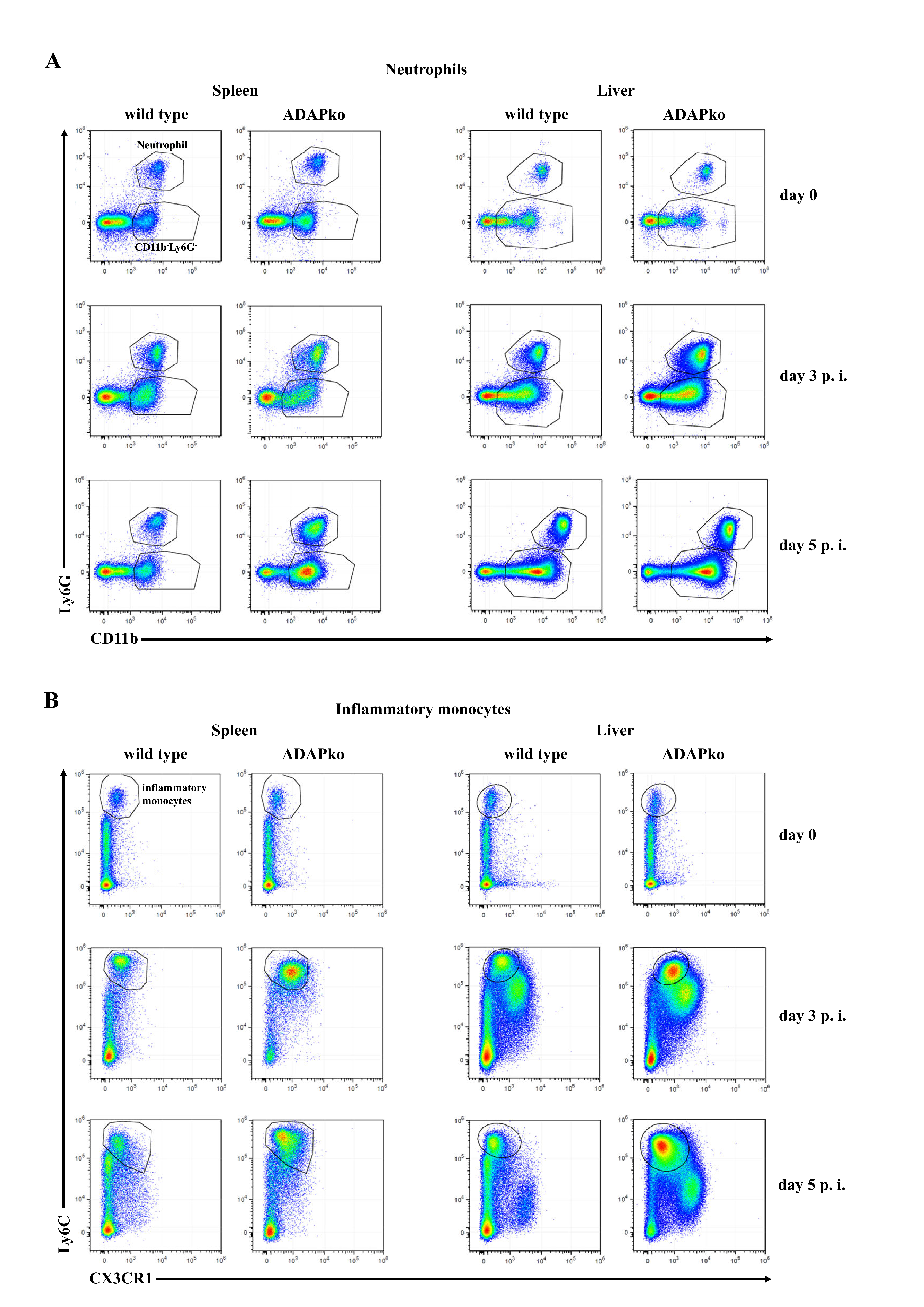

Supplement: Supplementary Figure 7 — ǀ FACS gating for neutrophils and inflammatory monocytes of wild type and ADAPko mice during Listeria monocytogenes infection. Representative gating scheme to define (A) neutrophils and (B) inflammatory monocytes from untreated (uninfected control mice, day 0) or Lm-infected (i. v. with 2.5 × 10^4^ CFU Lm) wild type and ADAPko mice at the indicated times post infection. Cells were pre-gated on leukocytes by means of FSC/SSC and doublets were excluded by FSC-A/FSC-H gating. Dead cells were eliminated by staining with live/dead marker and only CD45+ as well as lineage negative cells were further characterized (pre-gating not shown). Neutrophils were gated as Ly6G+CD11b+ cells and inflammatory monocytes as Ly6C+CX3CR1high. [file Image_7.tiff]
